# A low CO_2_-responsive mutant of *Setaria viridis* reveals that reduced carbonic anhydrase limits C_4_ photosynthesis

**DOI:** 10.1093/jxb/erab039

**Published:** 2021-02-02

**Authors:** Jolly Chatterjee, Robert A Coe, Kelvin Acebron, Vivek Thakur, Ragothaman M Yennamalli, Florence Danila, Hsiang-Chun Lin, Christian Paolo Balahadia, Efren Bagunu, Preiya P O S Padhma, Soumi Bala, Xiaojia Yin, Govinda Rizal, Jacqueline Dionora, Robert T Furbank, Susanne von Caemmerer, William Paul Quick

**Affiliations:** 1 C4 Rice Centre, International Rice Research Institute (IRRI), Los Baños, Philippines; 2 CSIRO Agriculture Flagship, Australian Plant Phenomics Facility, GPO Box 1500, Canberra, ACT 2601, Australia; 3 Department of Systems & Computational Biology, School of Life Sciences, University of Hyderabad, Hyderabad-500046, India; 4 Department of Bioinformatics, School of Chemical and Biotechnology, SASTRA Deemed to be University, Thanjavur, Tamilnadu-613401, India; 5 ARC Centre of Excellence for Translational Photosynthesis, Research School of Biology, Australian National University, GPO Box 1500, Canberra, ACT 2601, Australia; 6 Department of Animal and Plant Sciences, University of Sheffield, Sheffield S10 2TN, UK; 7 University of Essex, UK

**Keywords:** Carbonic anhydrase, C_4_ photosynthesis, C_4_ rice, forward genetics, mutant screen, *Setaria viridis*

## Abstract

In C_4_ species, β-carbonic anhydrase (CA), localized to the cytosol of the mesophyll cells, accelerates the interconversion of CO_2_ to HCO_3_^–^, the substrate used by phospho*enol*pyruvate carboxylase (PEPC) in the first step of C_4_ photosynthesis. Here we describe the identification and characterization of *low CO*_*2*_*-responsive mutant 1* (*lcr1*) isolated from an *N*-nitroso-*N*-methylurea- (NMU) treated *Setaria viridis* mutant population. Forward genetic investigation revealed that the mutated gene *Sevir.5G247800* of *lcr1* possessed a single nucleotide transition from cytosine to thymine in a β-CA gene causing an amino acid change from leucine to phenylalanine. This resulted in severe reduction in growth and photosynthesis in the mutant. Both the CO_2_ compensation point and carbon isotope discrimination values of the mutant were significantly increased. Growth of the mutants was stunted when grown under ambient *p*CO_2_ but recovered at elevated *p*CO_2_. Further bioinformatics analyses revealed that the mutation has led to functional changes in one of the conserved residues of the protein, situated near the catalytic site. CA transcript accumulation in the mutant was 80% lower, CA protein accumulation 30% lower, and CA activity ~98% lower compared with the wild type. Changes in the abundance of other primary C_4_ pathway enzymes were observed; accumulation of PEPC protein was significantly increased and accumulation of malate dehydrogenase and malic enzyme decreased. The reduction of CA protein activity and abundance in *lcr1* restricts the supply of bicarbonate to PEPC, limiting C_4_ photosynthesis and growth. This study establishes *Sevir.5G247800* as the major CA allele in *Setaria* for C_4_ photosynthesis and provides important insights into the function of CA in C_4_ photosynthesis that would be required to generate a rice plant with a functional C_4_ biochemical pathway.

## Teaser

Carbonic anhydrase is essential for C_4_ photosynthesis in *Setaria viridis.*

## Introduction

C_4_ plants have evolved a combination of anatomical and biochemical specializations that concentrate CO_2_ around Rubisco (Hatch, 1987). In C_4_ leaves, specialized mesophyll cells (MCs) and bundle sheath cells (BSCs) support a biochemical CO_2_ pump (von Caemmerer and Furbank, 2003) where each molecule of CO_2_ entering the cytosol of the MCs is converted to bicarbonate (HCO_3_^–^) by the activity of carbonic anhydrase (CA). This is then incorporated into phospho*enol*pyruvate (PEP) by PEP carboxylase (PEPC), yielding the C_4_ acid oxaloacetate (OAA). In most agriculturally important C_4_ species, the OAA is taken up into the chloroplast of the MCs where it is reduced to malate by NADP-dependent malate dehydrogenase (NADP-MDH). Malate is exported back to the cytosol and then diffuses into the BSCs. It is then transported into the chloroplast and oxidatively decarboxylated by NADP-dependent malic enzyme (NADP-ME), yielding CO_2_, NADPH, and pyruvate. CO_2_ is assimilated by Rubisco and pyruvate is transported into the chloroplast of the MCs where it is converted to PEP by pyruvate phosphate dikinase (PPDK). This pump can elevate CO_2_ in this compartment by up to 10-fold above air levels (Furbank and Hatch, 1987; von Caemmerer and Furbank, 2003), suppressing photorespiration and CO_2_ saturating photosynthesis in air.

The efficiencies of C_4_ photosynthesis have long driven interest in engineering a C_4_ photosynthetic pathway into C_3_ crop species (Matsuoka *et al.*, 2001; Häusler *et al.*, 2002; Miyao, 2003; Kajala *et al.*, 2011; Miyao *et al.*, 2011). In rice, this could potentially lead to increases in radiation use efficiency and yield of up to 50% (Hibberd *et al.* 2008). The C_4_ Rice Consortium (https://c4rice.com) is currently investigating the feasibility of engineering the leaf anatomy and biochemistry required to support a two-celled photosynthetic pathway (Kajala *et al.*, 2011; Lin *et al.*, 2016). To help achieve this, a toolkit of genes is being assembled. Unknown genes are being identified through forward genetics, whereby large populations of C_4_ plants are mutagenized and then screened for a loss of C_4_ photosynthetic characteristics and function (Furbank *et al.*, 2009). This approach has already led to the identification of some promising mutants with altered leaf anatomy (Rizal *et al*., 2015, 2017). However, there remains much which is unknown about the efficiencies, genes, and mechanisms underpinning the C_4_ photosynthetic process. Among these are the genetic components modulating photosynthetic cellular specialization (suberization of the BSC wall and positioning of the chloroplast), localization of C_3_ and C_4_ photosynthetic enzymes (C_4_ biochemistry, the Calvin cycle, and the photorespiratory cycle), the identity of transporters supporting C_4_ metabolic flux, and the regulators of C_4_ gene expression. Knowledge of these processes is an essential requirement for engineering a functional C_4_ photosynthetic pathway into rice.

In an earlier study, we described the development of high-throughput screening for identifying CO_2_-responsive mutants of the C_4_ monocot model species *Setaria viridis* (L.) P. Beauv. (Coe *et al.*, 2018). The mutant population was generated using *N*-nitroso-*N*-methylurea (NMU). There, we could identify 46 candidate mutant lines with reduced *F*_v_/*F*_m_ relative to wild-type (WT) plants under conditions of low CO_2_ partial pressure (*p*CO_2_) at 15 µbar (0.0015%). This threshold level was determined considering the difference in the CO_2_ compensation point in C_3_ and C_4_ species. Reduction in *F*_v_/*F*_m_ with an increased CO_2_ compensation point is used as a signature for decreased photosynthetic efficiency and a loss of C_4_ function.

In 1990, Hatch and Burnell (1990) demonstrated that *in vivo* CA activities in C_4_ leaves were only just sufficient to support rates of PEP carboxylation. Since then, it has been debated whether high CA activities are a requirement for evolution of C_4_ photosynthesis. The enzyme is a part of a multiple gene family, where one gene may give rise to multiple CA isoforms via alternative splicing of the coding sequence (CDS). Functional evaluation of any of these genes gives different phenotypic expression in different species, which makes it difficult to interpret its role unambiguously. Whether the CA activity in C_4_ leaves has been reduced by transgenesis or other genetic means; conflicting reports have suggested either a large inhibition of photosynthesis (von Caemmerer *et al.*, 2004) or that little or no effect was observed (Studer *et al.*, 2014; Osborn *et al.*, 2017). In this study, by adopting a forward genetics approach, we identify the CA allele responsible for the carbon-concentrating mechanism (CCM) in the C_4_ model species *S. viridis* to be the underlying causal gene of the *low CO*_*2*_*-responsive* mutant *lcr1* (NM04534 in Coe *et al.*, 2018) using next-generation sequencing and mapping. We study in detail the role of CA in C_4_ photosynthesis, plant growth, and isotope discrimination in *S. viridis*. This work provides insights into the function and genetic regulation of C_4_ photosynthesis.

## Materials and methods

### Plant material

A *low CO*_*2*_*-responsive* mutant (NM04534, *lcr1*) was identified from a population of *S. viridis* A10.1 NMU mutants as described in Coe *et al.* (2018). The mutant was identified based on its reduced *F*_v_/*F*_m_ values in ambient (390 µbar *p*CO_2_) and in low CO_2_ conditions (15 µbar *p*CO_2_). The *F*_v_/*F*_m_ values of the successive generations of *lcr1* were checked in ambient and low CO_2_ conditions using a PlantScreen Compact System (Photon Systems Instruments) machine with replicated progenies to finally achieve a stable phenotype in the M_5_ generation. A single M_5_ mutant line (progeny number 23-50) was selected and advanced for further characterization and gene identification. The mutant is characterized for its photosynthetic, leaf anatomy, and biochemical characters mostly on the plants of the M_5_ and M_6_ generation, except the *F*_v_/*F*_m_ value, which was measured throughout as a marker to select the true candidate mutants. The seeds of *S. viridis* A10.1 were obtained from Dr Thomas Brutnell (Danforth Plant Science Center), and the rice seeds were obtained from the genebank of the International Rice Research Institute (IRRI), Philippines.

### Plant growth conditions

Plants were grown in two different locations, at the IRRI, Los Banos, Philippines (14.1699°N, 121.2441°E); and at the Australian National University (ANU), Canberra, Australia (35.2809°S, 149.1300°E) in different CO_2_ levels. At IRRI, the plants were grown in low CO_2_ (15 µbar *p*CO_2_), ambient (390 µbar *p*CO_2_), and elevated *p*CO_2_ level 1 conditions (1% or 10 000 µbar *p*CO_2_), whereas in ANU the plants were grown in elevated CO_2_ level 2 conditions—at 2% CO_2_ (20 000 µbar *p*CO_2_)_._

Seed dormancy was broken by soaking in 5% liquid smoke (Wright’s Liquid Smoke, B&G Foods Inc., Roseland, NJ, USA) for 24 h; they were then thoroughly rinsed with water and sown on moist filter paper. When the hypocotyl was ~5 mm long, seedlings were transferred to 0.5 litre pots containing sterilized soil from the IRRI upland farm mixed with 0.4 g l^–1^ Osmocote Plus 15-9-12 (The Scotts Company Ltd, Thorne UK). Plants were cultivated in a greenhouse at ambient CO_2_ or inside custom-made growth chambers at different controlled CO_2_ concentrations, as mentioned above. Irradiance in the greenhouse ranged between 500 µmol m^–2^ s^–1^ and 2000 µmol m^–2^ s^–1^ with a day length of between 11 h and 13 h. Day and night temperature ranged between 21 °C and 34 °C, with a relative humidity of between 60% and 70%. Inside the growth chambers, ~63% of the ambient solar irradiance is transmitted at the canopy level; the air temperature was maintained at ~30 °C and a relative humidity of 60–70%.

At the ANU, seeds were germinated in garden soil mix fertilized with Osmocote (Scotts, Australia) in small containers before being transferred to individual 2 litre pots. Plants were always grown in controlled-environment chambers at an elevated CO_2_ level 2 (i.e. 2% CO_2_), irradiance 500 µmol photons m^–2^ s^–1^, 16 h photoperiod, and day and night temperature of 28 °C and 24 °C, respectively. Pots were watered daily.

### Chlorophyll fluorescence imaging

Chlorophyll fluorescence images were acquired with the PlantScreen™ Compact System (Photon System Instruments) as described in detail in the previous report by Coe *et al.* (2018). Images were analysed using Fluorcam 7 v1.024.2 (Photon Systems Instruments), and fluorescence parameters were calculated in the software according to Genty *et al.* (1989). Plants are imaged for *F*_v_/*F*_m_ after 30 min of dark adaptation, and data were used for candidate selection, checking heritability of the low *F*_v_/*F*_m_ character in the mutant, confirmation of BC_1_F_1_, and BC_1_F_2_ population screening. The maximum quantum efficiency of PSII photochemistry (*F*_v_/*F*_m_), which gives a good understanding of a plant’s photosynthetic efficiency, was calculated as: (*F*_m_–*F*_o_)/*F*_m_. Detailed kinetic studies were performed on the M_5_ generation mutant plants and the WT. Measurements of rapid chlorophyll fluorescence kinetics were made following acclimation for 20 s at six different actinic light intensities between 25 µmol m^–2^ s^–1^ and 1000 µmol m^–2^ s^–1^. Plants were characterized for the maximum efficiency of PSII in the light if all centres were open (*F*_v_′/*F*_m_′), (*F*_m_′–*F*_o_′)/*F*_m_′; PSII operating efficiency (ΦPSII), (*F*_m_′–*F*′/*F*_m_′); and photochemical (qP) and non-photochemical quenching (NPQ). qP (*F*_m_′–*F*′)/(*F*_m_′–*F*_o_′), relates PSII maximum efficiency to operating efficiency; and NPQ [(*F*_m_/*F*_m_′)–1] estimates the rate constant for heat loss from PSII. *F* and *F*′ are the fluorescence emission from a dark- or light-adapted leaf; *F*_o_ and *F*_o_′ are the minimal fluorescence from a dark- and light-adapted leaf; *F*_m_ and *F*_m_′ are maximal fluorescence from a dark- and light-adapted leaf, respectively; and *F*_v_ and *F*_v_′ are the variable fluorescence from a dark- and light-adapted leaf. Details of the method are given in Coe *et al.* (2018). Fluorescence values represent the median of fluorescence values obtained for each plant and nine plants per line.

### Plant biomass

The number of tillers was counted and plant height was measured at the maximum tillering stage taking measurements from the soil surface to the tip of the youngest fully expanded leaf prior to destructive biomass measurements. All above-ground biomass (leaves, stems, and sheaths) was harvested, weighed, placed in papers bags, and oven-dried at 70 °C until a constant dry biomass weight was achieved. Leaf chlorophyll content was estimated at the mid-tillering stage using the upper fully expanded leaves employing a SPAD Chlorophyll Meter (SPAD, Konica Minolta, Japan). Values given are the average ±SD of 10 plants per line.

### Leaf microscopy

Leaf anatomy measurements were scored from the three cleared transverse leaf section images on the middle portion of one leaf each taken from five plants per line. Cleared leaf sections and fresh leaf sections are prepared and imaged as described in Chatterjee *et al.* (2016). The sections were imaged by an Olympus BX51 compound microscope using bright field for cleared sections and an Olympus BX52 disc spinning fluorescent microscope for fresh sections. The images were acquired with an Olympus DP71 camera. These were analysed with Olympus CellSens software (http://www.olympus-lifescience.com) and ImageJ software v.1.43 (Schindelin *et al.* 2015) to calculate leaf thickness (µm), BSC (µm^2^), MC (µm^2^), and epidermal cell (µm^2^) area, and area of the chloroplast in BSC area (µm^2^).

### Stomatal measurement

Stomatal measurements were performed on a total of five leaves taken from five plants per line. Leaf nail varnished images of abaxial imprints were used to score stomatal density and stomatal size under ×20 and ×40 magnification with the BX51 Olympus bright field microscope. Stomatal density was counted from 25 random observations and the length and width were determined from observation of 25 random stomata per line.

### Photosynthetic measurements

Gas exchange measurements were performed on M_6_ generation plants. Leaf gas exchange measurements were made using a Li-6400XT infrared gas analyzer (LI-COR Biosciences) on the plants grown at ambient or in 1% CO_2_ (elevated CO_2_ level 1). The cuvette was fitted with a standard 2×3 cm leaf chamber and a 6400-02B light source. Measurements were made after the leaves were acclimated in the cuvette for ~30 min, with a constant air flow rate of 400 μmol s^–1^, leaf temperature of 30 °C, leaf to air vapour pressure deficit between 1.0 kPa and 1.5 kPa, and relative humidity of 60–65%. Data were acquired between 08.00 h and 13.00 h. Measurements were made on at least three plants during the tillering stage on the mid-portion between the proximal and distal end of a fully expanded leaf. The response curves of the net CO_2_ assimilation rate (*A*, µmol m^–2^ s^–1^) to changing intercellular *p*CO_2_ (*C*_i_, µbar) were acquired by first stabilizing the plants in 400 µbar *p*CO_2_ and then increasing the *C*_a_ (*p*CO_2_ in the cuvette) stepwise from 20 µbar to 2000 µbar at a photosynthetic photon flux density (PPFD) of 1800 µmol photon m^–2^ s^–1^ at an appropriate O_2_ level (2, 21, 40, 60, and 80% O_2_). The CO_2_ compensation point (Γ) and maximum carboxylation efficiency (CE) were calculated from the intercept (Vogan *et al.*, 2007) and slope (Wang *et al.*, 2006) of the CO_2_–response curves. Light–response curves were acquired by increasing the PPFD from 0 to 2000 µmol photon m^–2^ s^–1^ at a *C*_a_ of 400 µbar. The quantum efficiency for CO_2_ assimilation (Φ) was calculated from the slope of the light–response curves (PPFD <100 μmol photons m^–2^ s^–1^). Dark respiration rate (*R*_d_) measurements were calculated at a *C*_a_ of 400 μbar.

### Gas exchange and isotopic discrimination

Carbon isotope discrimination was determined by combusting dry leaf tissue utilizing a Eurovector elemental analyser using plants grown in ambient *p*CO_2_ at IRRI. The three youngest fully expanded leaves on the main stem of three different 25 days-old-plants were sampled and dried in an oven at 70 °C, ground into a fine powder (Precellys 24, Bertin Instruments, France), and 1.1–1.3 mg of leaf sample was sent to the Analytical Service Laboratory at the IRRI for quantification of dry carbon isotope. The ratio of ^13^C to ^12^C (δ) is reported relative to the Pee Dee Belemnite standard (Craig, 1957). Based on the equation from Farquhar *et al.* (1989), the carbon isotope composition (δ _p_) was calculated as:

$$\begin{array}{l} \delta p=\frac{R_{P}-R_{S}}{R_{S}}=\frac{R_{P}}{R_{S}}-1 \end{array}$$

where, *R*_S_ is the molar abundance ratio (^13^C/^12^C) of the PDB standard, and *R*_P_ is the molar abundance ratio (^13^C/^12^C) of a plant sample. Carbon isotope discrimination (∆, ‰) was calculated as:

$$\begin{array}{l} \Delta =\frac{R_{a}}{R_{p}}-1=\frac{\delta_{a}-\delta_{p}}{1+\delta_{p}} \end{array}$$

where *R*_a_ is the molar abundance ratio (^13^C/^12^C) in air, and δ _a_ is the ratio of ^13^C/^12^C of free atmospheric CO_2_ on the PDB scale and is approximately –8‰ according to the Earth Systems Research Laboratory.

Isotopic discrimination was also measured in combination with gas exchange measurement using tunable diode laser (TDL) spectroscopy as described by Evans and von Caemmerer (2013) for ^13^C isotope discrimination and as described by Osborn *et al.* (2017) for C^18^O^16^O discrimination. Gas exchange and carbon isotope discrimination measurements were made using a 6 cm^2^ chamber of the LI-6400 with a red/blue light-emitting diode (LED) light source (Li-Cor, Lincoln, NE, USA). Two LI-6400 chambers and the plants were placed in a temperature-controlled cabinet with fluorescent lights (TRIL1175, Thermoline Scientific Equipment, Smithfield, Australia). The CO_2_ in the leaf chamber was set at 380 µmol mol^–1^, flow rate at 200 µmol s^–1^, and irradiance at 1500 µmol quanta m^–2^ s^–1^. Leaf temperature was controlled at 25 °C. O_2_ [2% in N_2_, mixed by mass flow controllers (Omega Engineering Inc., Stamford, CT, USA)], was supplied to the LI-6400s after humidification of the air by adjusting the temperature of water circulating around a Nafion tube (Perma Pure LLC, Toms River, NJ, USA, MH-110-12P-4). For ^13^C discrimination measurements, the gas exchange was coupled to a TDL (TGA100a, Campbell Scientific, Inc., Logan, UT, USA). Measurements were made at 4 min intervals for 20 s, with 10–12 measurements per leaf, and the last five measurements were averaged. The measurements of C^18^O^16^O discrimination were made as described by (Osborn *et al.*, 2017). Simultaneous measurements of CO_2_, H_2_O, C^18^O^16^O, and H_2_^18^O were made by coupling two LI-6400XT gas exchange systems to a TDL (model TGA200A, Campbell Scientific Inc.) and a cavity ring-down spectrometer (L2130-i, Picarro Inc., Sunnyvale, CA, USA) to measure the oxygen isotope composition of water vapour. The system is essentially that described above except that the TGA100a was replaced by a TGA200A and the additional laser for water vapour measurements was added together with a 16 port distribution manifold. The sample and reference gas streams were sampled with a T junction in the match valve tubing and in the reference line of the LI-6400XT, respectively. This allowed leaves of two plants to be measured in sequence, with each LI-6400XT sampled by the TDL at 4 min intervals for 20 s at the sample and reference line. The Picarro cavity ring-down spectrometer sampled for 3 min, so that leaves were sampled at 6 min intervals. Gas exchange was calculated using the equations presented by von Caemmerer and Farquhar (1981), and Δ was calculated from the equation presented by Evans *et al.* (1986). The measurements were performed on 5-week-old plants grown at 2% CO_2_ at the ANU. Measurements were made at 2% O_2_, 380 µmol mol^–1^ CO_2_, leaf temperature of 25 °C, irradiance of 1500 µmol quanta m^–2^ s^–1^, and relative humidity of 55%. Each leaf was measured at 4 min intervals, and 10 readings were taken.

### Generation of the segregating population and whole-genome sequencing of bulked segregants

The M_5_ progeny of *lcr1* was crossed with WT plants, the latter as pollen donor, to obtain the BC_1_F_1_ generation (following the protocol of Jiang *et al.*, 2013). BC_1_F_1_ lines were checked for a recovery of the *F*_v_*/F*_m_ value in true F_1_, and then selfed to produce the BC_1_F_2_ segregating population, which was used for whole-genome sequencing. From a BC_1_F_2_ population of 300 plants, equal quantities of genomic DNA were pooled from 45 individuals exhibiting the *lcr* phenotype (homozygous mutant pool) and 54 individuals without the *lcr* phenotype (azygous pool). Total genomic DNA was extracted from young leaves of a mid-tillering plant using the CTAB (cetyltrimethylammonium bromide) protocol (Murray and Thompson, 1980). Genomic DNA was also pooled from 50 WT plants (WT pool). These pooled samples were sequenced using Illumina HiSeq2000 platforms at Beijing Genome Institute BGI Tech Solutions (Hongkong) Co. Ltd, Shenzhen, China. The number of paired-end reads generated of size 125 bp was ~108.1 million for the WT pool, ~91 million for the azygous pool, and ~107.3 millions for the mutant pool, yielding 23–33 Gb of sequence data with a genome coverage of 45—65x for each.

### Candidate gene discovery

The raw sequence data were processed so that reads with poor quality bases were either trimmed or filtered using Trimmomatic v0.32 (Bolger *et al.*, 2014). The minimum Phred quality score was set to 20, and reads containing partial/complete Illumina adaptor sequences were removed. The processed reads were aligned against the *S. viridis* reference genome (version 1.1 from the Phytozome database) using the BWA aligner (version 0.7.12; Li, 2013, Preprint). The alignment was improved by discarding the duplicate read pairs using the PICARD tool (https://broadinstitute.github.io/picard/), and the base quality was re-calibrated with the help of the highest quality variants, present within the sequence data itself, as a gold standard variant set, using the Genome Analysis Tool Kit (GATK-3.7-0; McKenna *et al.*, 2010). Variant calling was also done by using the same tool such that alleles were first called at all genomic positions, followed by variant calling (DePristo *et al.*, 2011).

The variants with an allele frequency of ≤0.3 were assumed to result from incorrect alignment of minority reads, and were thus removed from the analysis. Only variants occurring with support of at least 10 reads, and a genotype quality (GQ) of ≥30, at least in the mutant pool, were considered for downstream analysis. Variant(s) associated with the mutant phenotype are likely to be common to all individuals of the mutant pool (yielding a mutant allele frequency of 1); however, such variants will either be absent or in a heterozygous state in the individuals of the azygous pool (yielding a mutant allele frequency of ~0.33). Such variants (mutant pool) are also likely to show a typical linkage pattern with the neighbouring variants in the mutant pooled sample. Therefore, genomic loci harbouring such variants were extracted from the mutant pool and were considered as candidates.

The candidate mutations were annotated using the *S. viridis* genome annotation available at the Phytozome database (release 12; https://phytozome.jgi.doe.gov/pz/portal.html). Mutations located within a gene leading to an alteration to protein sequence became the basis to identify candidate genes.

### Functional characterization of amino acid substitution by sequence conservation and protein structural analysis

In order to examine the effect of Leu156Phe substitution, the amino acid conservation profile was first obtained. Members of the ‘Carbonic anhydrase 2’ gene family from Angiosperms were obtained from the Phytozome database (https://phytozome.jgi.doe.gov), available under the ‘Gene ancestry’ tab of the *Sevir.5G247800* information page. Using the Biomart tool of Phytozome, 226 peptide sequences were extracted and multiple-aligned using the MAAFT tool (version 7; https://mafft.cbrc.jp/alignment/server/; Mode: L-INS-i). Fifteen sequences, which appeared partial/incomplete, were dropped and the fully conserved residues were finally identified from the multiple alignment itself.

The mechanism of β-CA function in plants had been thoroughly examined in the β-CA protein of *Pisum sativum* by Kimber and Pai (2000), wherein the residues were identified for Zn binding, active site, catalytic cleft formation, non-catalytic bicarbonate-binding pocket, and solvent access. To further explore the role of the residues in β-CA function, and loss of function due to Leu156Phe substitution, structural models were generated and analysed. The native and mutant peptide sequence of Sevir.5G247800 was given as input to the Rosetta tool to build 3-D protein structures using the comparative modelling approach (Kim *et al.*, 2004). Rosetta provided five models with a confidence score ranging from 0.0 to 1.0, where a confidence score of 0.0 is indicative of a bad model and 1.0 of a perfect model (Song *et al.*, 2013).

Among the five models, the structure present in the same conformation as in Type I CA (based on the classification of Rowlett, 2010) was selected for further analysis of (i) contact order (CO) calculation; (ii) estimation of long-range interacting residues by identifying the primary, secondary, and tertiary shell of residues; and (iii) analysing the dimer interface of native and mutant proteins. The CO quantifies the global protein topology and correlates with protein folding rates (Plaxco *et al.*, 1998); thus, by comparing intraprotein interactions of the native and mutant proteins, the stabilizing forces can be quantified. The higher the CO value, the higher the stabilizing factor of the interaction type (Rader *et al.*, 2012).

As a complementary method of identifying short- and long-range interactions, inspiration came from electron shells surrounding the nucleus. In the case of proteins, residue 156 was considered as the nucleus, and the residues interacting with 156 (Leu or Phe) were considered as the first shell of interacting residues; the residues that interact with these first shell of interacting residues were considered as the second shell of interacting residues, and similarly the residues that interact with the second shell of interacting residues were identified as the third shell of interacting residues (Drawz *et al.*, 2009; Brodkin *et al*., 2011, 2015) In each shell, redundancy was removed and directionality of moving away from the nucleus was maintained.

The third analysis involved determining the residues interacting between the monomers of Sevir.5G247800. This directly established the various unique interactions in native and mutant proteins that stabilize the dimer stoichiometry of Type I CA. This analysis was performed on three protein structures; the homodimer of the native, the homodimer of the mutant, and a heterodimer made of β-CA isoforms (Sevir.5G247800 and Sevir.5G247900.1). This was done so that an isoform was hypothesized to interact with other isoforms, leading to re-establishment of the lost function due to mutation at position 156.

### Candidate gene expression analysis

Progenies of the M_6_ generation were used for gene expression analysis. Plants were grown in ambient CO_2_ conditions in IRRI. The youngest fully expanded third leaves were harvested for total RNA extraction using TRIZOL (Invitrogen). Total RNA was treated with RQ1 RNase-free DNase (Promega). DNase-treated samples were reverse transcribed using the First-strand cDNA synthesis kit (Roche Diagnostics). cDNA samples were normalized to 100 ng µl^–1^ for all the quantitative PCR (qPCR) experiments using a NanoDrop 8000 spectrophotometer (ThermoFisher Scientific). LightCycler 480 SYBR Green I Master mix (Roche Diagnostics) was used as dye for the qPCR run on StepOnePlus Real-Time PCR (ThermoFisher Scientific). Three biological replicates for the WT and mutant line were obtained. Relative gene expression was computed using the 2^–ΔΔCt^ method (Livak and Schmittgen, 2001; Schmittgen and Livak, 2008) with polylpolyglutamate synthase (FGS; Sv035045) and glyceraldehyde 3-phosphate dehydrogenase (GAPDH; Sevir.1G225700) as internal control genes with primer sequences obtained from Lambert-Frotte (2015). Amplification specificities of all primers used were analysed using melt-curve analysis on each qPCR run. Primer sequences used were: 5′-GCGGGTGCCTTTGCCTCCCT and 5′-CTGGGTGCCTGGCCCTCCT for *Sevir.5G247800.1*, 5′-TGCTTCGCTGGGTTGAGCATCGT and 5′-CTGGGTGCCT GGCCCTCCT for *Sevir.5G247800.2*, 5′-CACCTTCTCCTCCACC GCACA and 5′-GCGATGTTGCGGACAGCGA for *Sevir.5G247900*, and 5′-CGCCGCGGGAGAACCCGA and 5′-CGGCCCGAACAG CATGGGGT for *Sevir.5G248000*. Transcript abundance for *Sevir.2G245200* was undetectable and so was not included in the analysis.

### Leaf protein and western blot

Progenies of the M_6_ generation were used for protein analysis. Leaf samples were harvested between 09.00 h and 11.00 h from the fourth fully expanded leaf of plants grown at ambient CO_2_ conditions at IRRI. Proteins were extracted by homogenizing leaf material in 250 µl of buffer containing 100 mM Bicine-KOH, pH 9.8, and 25 mM DTT (Lin *et al.*, 2016). Proteins were fractionated by 12% (w/v) SDS–PAGE with Precision Plus Protein™ Unstained Standards (BIORAD, USA) used as the loading control. Samples were loaded based on equal leaf weight [34 µg for PEPC and Rubisco large subunit (LSU), 136 µg for PPDK, and 340 µg for MDH, ME, and CA]. After electrophoresis, proteins were electroblotted onto a polyvinylidene difluoride membrane and probed with rabbit antisera containing antibodies made against PEPC protein, MDH protein, ME protein, Rubisco LSU protein (all provided by Richard Leegood, University of Sheffield, UK), PPDK protein (provide by Chris Chastain, Minnesota State University, USA), and CA protein (provided by Jim Burnell, James Cook University, Australia). The dilutions of PEPC, PPDK, MDH, ME, Rubisco LSU, and CA antibodies were 1:20 000, 1:20 000, 1:5000, 1:5000, 1:30 000, and 1:5000, respectively. A peroxidase-conjugated secondary antibody (Sigma Aldrich, USA) was used at a dilution of 1:5000, and immunoreactive bands were visualized with ECL Western Blotting Detection Reagents (GE Healthcare, UK). Total soluble leaf protein content was determined using the Bradford method (Bradford, 1976).

### Enzyme activity

 Enzyme activities were measured on 5-week-old plants that were grown in 2% CO_2_ conditions (elevated CO_2_ level 2). A high CO_2_ concentration was used to obtain sufficient healthy leaves of mutants to perform the assays. The activities of CA, Rubisco, and PEPC were determined as described by Osborn *et al.* (2017).

### Statistical analysis

 The significant differences in the results of the WT and the mutant were obtained by statistical analysis performed in R version 3.0.0 (The R Foundation for Statistical Computing, Vienna, Austria) or OriginPro 9.1 (OriginLab Corporation) using a one-way ANOVA and a least significant difference test or a two-sample *t*-test.

## Results

### Identification of the *Setaria* mutant responsive to low *p*CO_2_

**Fig. 1.**
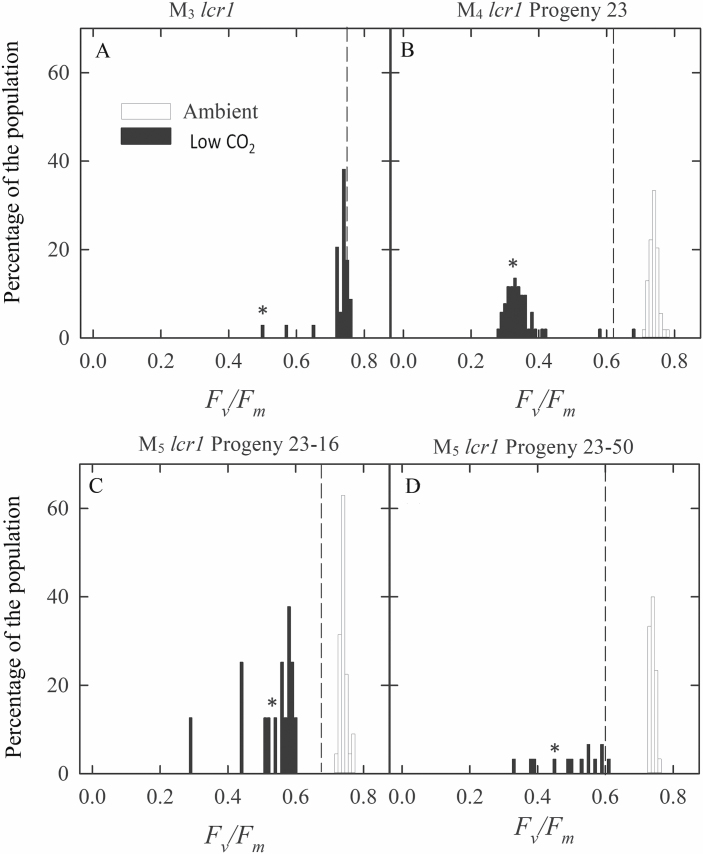
Identification of the mutant *lcr1*. Histogram of *F*_v_/*F*_m_ at ambient *p*CO_2_ (390 μbar) and after 48 h at low *p*CO_2_ (15 μbar). M_3_ (A), M_4_ (B), and two M_5_ generation progenies (C, D); *n*=80, 100, and 50 for *lcr1* at the M_3_, M_4_, and M_5_ generations, respectively. Dashed vertical lines are the average *F*_v_/*F*_m_ of wild-type *Setaria viridis* at low CO_2_ for that batch (*n*= 260, 100, and 50 for M_3_, M_4_, and M_5_ respectively). Asterisks show the bin from which the progeny advanced to each generation were selected.

**Fig. 2.**
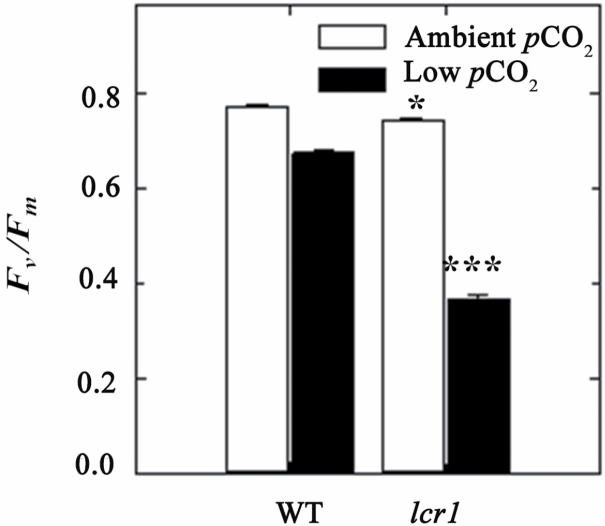
Chlorophyll fluorescence properties of *lcr1*. The graph shows the *F*_v_/*F*_m_ of wild-type (WT) and M_5_ generation *lcr1* plants grown at ambient and low *p*CO_2_. Values are the average ±SE of 10 individual plants per line. Significant variation in values of the WT and *lcr1* are denoted with *P<0.05 and ***P<0.001.

The *lcr1* mutant was identified from a screening of the *F*_v_*/F*_m_ response to low CO_2_ of 7900 NMU mutant lines. A cut-off value of 0.6 QY of PSII (*F*_v_*/F*_m_) was set to identify the low CO_2_ mutants, as we previously established that the *F*_v_*/F*_m_ value decreased to 0.6 in WT *Setaria* after 48 h of low CO_2_ treatment (Coe *et al.*, 2018). Initially two individuals of M_3_ progeny of the mutant line were identified with a reduced *F*_v_*/F*_m_ relative to WT plants at low *p*CO_2_ (Fig. 1A). Its progeny in M_4_ inherited mutant-like *F*_v_*/F*_m_ values (<0.6) after the low CO_2_ treatment (Fig. 1B). By the M_5_ generation, a stable *F*_v_*/F*_m_ response to low *p*CO_2_ was observed (Fig. 1C, D), which was very distinct from the WT response and always in the range of the mutant category. Two of the M_5_ lines were studied further for detailed characterization. The *lcr1* mutant exhibited heritable reductions of ~50% in *F*_v_*/F*_m_ after low *p*CO_2_ treatment for 48 h (Fig. 2). This response was significantly different from that of the WT, where the values were only 25% less after the treatment. The *F*_v_*/F*_m_ values of *lcr1* were only slightly reduced under normal *p*CO_2_ conditions.

### *lcr1* showed reduction in overall growth at ambient CO_2_, but recovered at high CO_2_

**Fig. 3.**
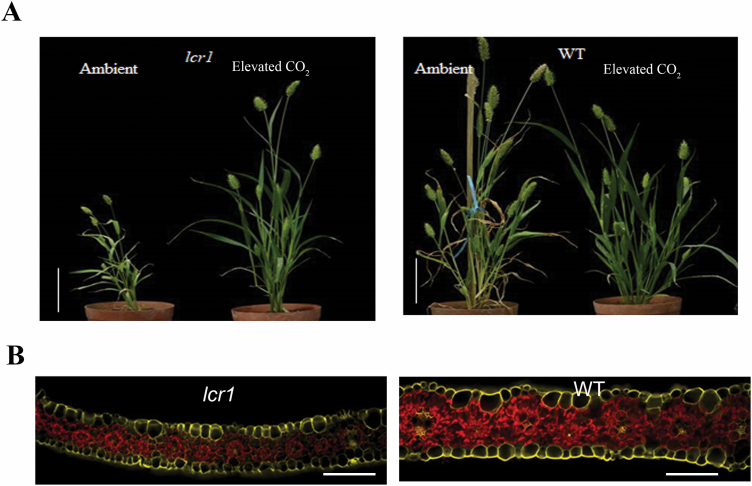
Plant morphology and leaf anatomy of *lcr1*. (A) The *lcr1* plant shows a dwarf phenotype in ambient *p*CO_2_, but recovered in elevated *p*CO_2_. Scale bar=5cm. (B) Leaves are thin in *lcr1* in ambient *p*CO_2_. Quantification of leaf anatomical traits is given in Table 1. Red shows autofluorescence of chloroplast; yellow shows the autofluorescence from the cell walls. Scale bar=50 µm. Plant images are redrawn from Coe *et al.* (2018).

When grown at ambient *p*CO_2_, the mutant plants were significantly shorter, had fewer tillers and panicles, and accumulated less fresh and dry biomass (Fig. 3A; Table 1). Plants were visually pale, and leaves were significantly thinner and had lower MC, BSC, and adaxial cell areas (Fig. 3B; Table 1). The chloroplast area in the BSCs was approximately half that of WT plants. However, when grown at elevated *p*CO_2_ (level 1), the mutant was almost indistinguishable from WT plants (Fig. 3A) and defects in leaf anatomy recovered significantly (Table 1; Supplementary Fig. S1). Plants grown in elevated CO_2_ level 2 also did not show any growth abnormalities. Stomatal number was significantly increased in mutant plants under ambient growth conditions, but were not different when grown at elevated CO_2_ levels 1 and 2 (Table 1; Supplementary Table S1)

**Table 1.** Morphological characteristics of *lcr1* in ambient and elevated CO_2_

Characteristics	Unit	Ambient *p*CO_2_		Elevated *p*CO_2_	
		WT	*lcr1*	WT	*lcr1*
*Biomass*					
SPAD	Relative unit	36.6±1.4 a	10.8±0.5 b***	42.9±0.5 a	41.6±0.5 a
Height	cm	31.9±1.0 b	12.0±0.6 c***	49.5±21.5 a	48.2±2.8 a
Tiller number	Count	6.7±0.6 b	4.3±0.3 c***	17.0±2.6 a	16.8±3.0 a
Panicle number	Count	5.2±0.4 a	3.1±0.3 b**	8.1±0.4 a	7.1±0.3 a
Whole plant FW (vegetative part)	g	6.83±0.4 a	4.61±0.3 b*	6.83±0.19 a	5.46±0.10.4 a
Whole plant DW (vegetative part)	g	0.58 ±0.64 a	0.4±0.3 b*	1.00±0.44 a	0.840±0.20 a
Leaf anatomy					
Leaf thickness	µm	135.3±5.0 a	86.2±2.7 b ***	133.8±3.1 a	136.2±1.9 a
Area of a single mesophyll cell	µm^2^	596.7±67.5	263.6±22.9 c***	626.2±116.6 a	457.7±37.8 *
Area of an individual bundle sheath cell	µm^2^	400. 2±39.8 a	188.6±14.7 c***	400.2±23.6 a	362.7±31.4 b*
Chloroplast area in bundle sheath cells	µm^2^	50.2±5.4 a	24.8±0.4 c***	58.9±5.0 a	48.2±6.8 b*
*Stomatal characteristics*					
Stomatal density	Count per mm^2^	71.1±6.5 a	103.9±9.4 b***	67.1±4.9 a	71.8±6.2 a
Stomatal length	µm	27.4±1.3 a	24.7±1.4 a	25.8±0.3 a	24.6±0.8 a
Stomatal width	µm	21.5±0.9 a	16.9±1.6 a	21.5±1.1 a	23.7±2.1 a

Biomass values are the average ±SD of 10 plants per line. Leaf anatomy scores are the average ±SE of at least five transverse leaf section images from each of five plants per line. Stomatal measurements are the average ±SE of scores taken on the abaxial side of five leaves from five different plants per line. Different letters denote statistical significance (***P<0.001, **P<0.01, *P<0.05) within a trait category. Plants were grown in either ambient (390 µbar) or 10 000 µbar *p*CO_2_.

### Physiological properties of *lcr1*

#### lcr1 *shows reduced quantum efficiency and photosynthesis in response to light*

**Fig. 4.**
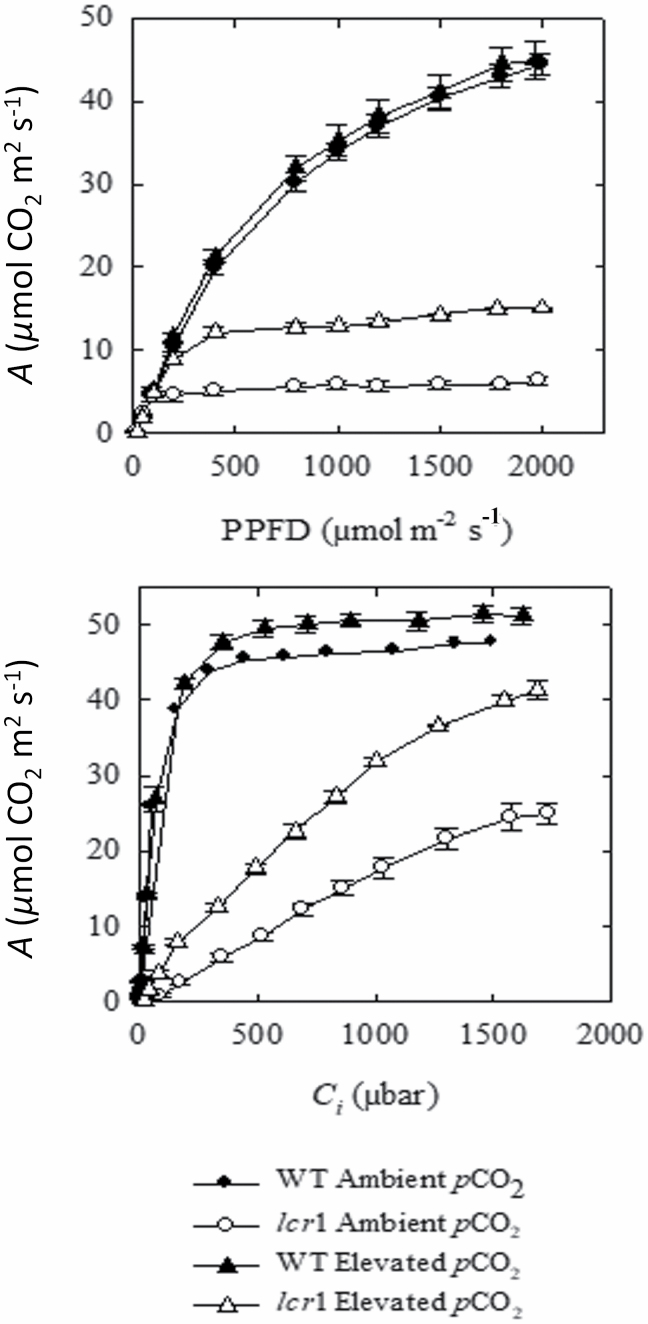
Photosynthetic response of *lcr1* in ambient *p*CO_2_ and elevated *p*CO_2_. (A) Net CO_2_ assimilation rate (*A*) in response to changes in photosynthetic photon flux density (PPFD) and (B) intercellular *p*CO_2_ (*C*_i_). Measurements were made at a *p*CO_2_ (*C*_a_) of 400 µbar or 1800 µmol photons m^–2^ s^–1^ and a leaf temperature of 30 °C. Values are the average ±SE of one fully expanded leaf from three different plants of the wild-type (WT, filled symbols) and M_6_ generation *lcr1* (open symbols) plants grown at ambient (circles) or elevated (10 000 ppm) *p*CO_2_ (triangles).

The CO_2_ assimilation rate (*A*) was severely affected in *lcr1*. In response to light, CO_2_ assimilation in the mutant was saturated at very low irradiance (~100 µmol m^–2^ s^–1^), whereas the WT plants exhibited a typical C_4_ response, with a curvilinear increase in CO_2_ assimilation rate in response to increasing irradiance (Fig. 4A). Values for Φ were also significantly reduced, suggesting that light utilization efficiency was affected (Table 2). Analysis of the rapid response of chlorophyll fluorescence kinetics of ambient-grown plants revealed that NPQ was increased in the mutant relative to WT plants between 200 µmol m^–2^ s^–1^ and 500 µmol m^–2^ s^–1^ (Supplementary Fig. S2a). Corresponding decreases were observed in the maximum efficiency of PSII in the light (Supplementary Fig. S2b), ΦPSII (Supplementary Fig. S2c), and qP (Supplementary Fig. S2d). We have seen recovery in light response of the mutant to some extent when grown in elevated CO_2_ conditions. When mutants were grown at elevated *p*CO_2_, CO_2_ assimilation rates were saturated at a higher irradiance (~400 µmol m^–2^ s^–1^), although the response was still uncharacteristic of a typical C_4_ photosynthetic response, compared with WT plants (Fig. 4A). No significant differences were observed in the values for Φ of these plants (Table 2).

**Table 2.** Photosynthetic response of *lcr1* in ambient and elevated CO_2_ conditions

	Γ	CE	*R*_d_	Φ
	µbar	mol m^–2^ s^–1^ bar^-1^	µmol CO_2_ m^–2^ s^–1^	mol CO_2_ mol^–1^ quanta
	Ambient *p*CO_2_			
WT	2.27±0.62 a	0.56±0.04 a	1.01±0.18 b	0.06±0.00 a
*lcr1*	66.16±11.53 c***	0.02±0.00 d***	1.46±0.19 a*	0.05±0.00 b*
	Elevated *p*CO_2_			
WT	2.57±0.19 a	0.58±0.05 b	1.15±0.1 b	0.06±0.00 a
*lcr1*	18.00±2.63 b**	0.05±0.00 c**	1.06±0.14 b	0.06±0.00 a

CO_2_ compensation point (Γ), carboxylation efficiency (CE), respiration rates (*R*_d_), and quantum yield for CO_2_ assimilation (Φ ). Measurements of Γ and CE were made at a PPFD of 1800 µmol photons m^–2^ s^–1^, and Φ at a *p*CO_2_ (*C*_a_) of 400 µbar and a leaf temperature of 30 °C. Values are the average ±SE of one leaf from 4–8 plants per line grown at ambient or elevated *p*CO_2_. Different letters denote statistically significant differences (***P<0.001, **P<0.01, and *P<0.05). Plants are grown in either ambient (390 µbar) or elevated (10 000 µbar) CO_2_ level 1.

#### lcr1 *showed reduced CO*_2_*assimilation in response to intercellular CO*_2_

In response to changes in intercellular *p*CO_2_ (*C*_*i*_), CO_2_ assimilation rates in mutant plants grown at ambient *p*CO_2_ increased linearly, reaching a semi-plateau at ~1600 µmol CO_2_ m^–2^ s^–1^ (Fig. 4B). Partial recovery in CO_2_ response was observed when the plants were grown at elevated CO_2_. At elevated *p*CO_2_ level 1, CO_2_ assimilation rates of *lcr1* plants were higher than in ambient levels at all intercellular *p*CO_2_, with a linear increase up to 1000 µbar. The carboxylation efficiency (CE) of mutants was significantly lower compared with WT plants at all growth *p*CO_2_. CO_2_ compensation points (Γ) were 66.16 µbar and 18.00 µbar in *lcr1* plants grown at ambient and elevated *p*CO_2_ compared with 2.57 µbar in WT plants. Rates of respiration were ~1.5 times higher in plants grown at ambient *p*CO_2_, but no significant differences were found for plants grown at elevated *p*CO_2_ (Table 2).

#### *Isotope discrimination is severely affected in* lcr1

**Fig. 5.**
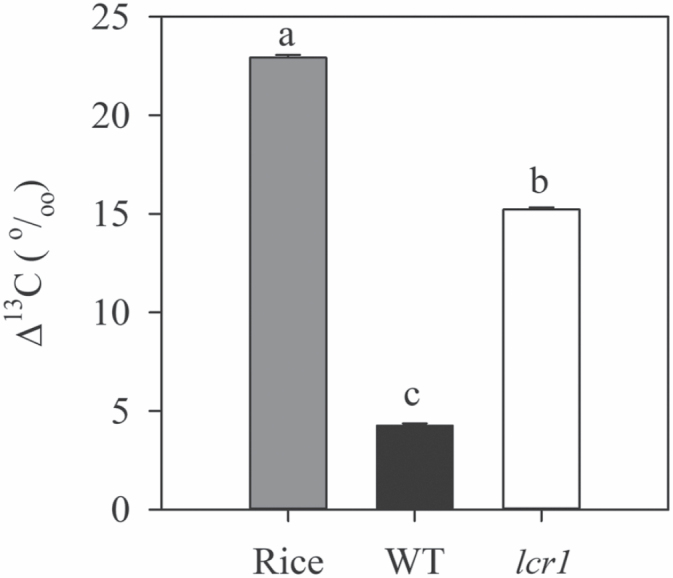
Leaf dry matter carbon isotope discrimination in *lcr1*. ∆ ^13^C value of rice, wild-type *Setaria viridis* (WT), and M_6_ generation *lcr1* plants. Values are the average ±SE of 10 plants per line. Different letters denote statistically significant differences (*P*<0.05).

Gas exchange measurements were repeated at ANU on the plants grown in high CO_2_ conditions (2% *p*CO_2_). The gas exchange measurement, coupled with a TDL trace gas analyser to measure ^13^C isotope and ^18^O (C^18^O^16^O) discrimination, showed similar results to the dry matter isotope measurements at IRRI, confirming a severe block of CO_2_ assimilation in the mutant (Table 3). Stomatal conductance remained either unaffected or slightly better in the mutant. The mutant showed a very high *C*_i_*/C*_a_ value because of low CO_2_ assimilation rates (Table 3). The exchange of ^18^O between CO_2_ and water is facilitated by the presence of CA in leaves, which catalyses the interconversion of CO_2_ and bicarbonate (HCO_3_^–^). The C^18^O^16^O discrimination is dependent on the *C*_i_/*C*_a_ ratio and CA activity. Although the C^18^O^16^O discrimination values of the WT and mutants were not significantly different, the values for the mutant were lower than expected at the given *C*_i_/*C*_a_ (Table 3). There is a significant increase in carbon isotope discrimination (∆ ^13^C) in *lcr1* at both ambient and high CO_2_ levels. The ∆ ^13^C value fell in between the range of ∆ ^13^C values of any of the C_3_ or C_4_ species when measured on the ambient CO_2_-grown plants (Fig. 5).

**Table 3.** Gas exchange measurements of *lcr1* on elevated CO_2_ level 2-grown *plants*

	Units	WT	*lcr1*
Gas exchange measurements with ^13^C laser			
CO_2_ assimilation rate	µmol m^–2^ s^–1^	33.8±1.2	13.7 1.2***
Stomatal conductance	mol H_2_O m^–2^ s^–1^	0.29±0.03	0.41±0.03*
Ratio of intercellular to ambient CO_2_, *C*_i_/*C*_a_	Ratio	0.46±0.04	0.82±0.01***
∆ ^13^C	‰	2.8±0.35	13.3±0.34***
Gas exchange measurements with C^18^O^16^O laser			
CO_2_ assimilation rate	µmol m^–2^ s^–1^	36.1±1.7	14.3±0.79***
Stomatal conductance	mol H_2_O m^–2^ s^–1^	0.31±0.03	0.39±0.02*
Ratio of intercellular to ambient CO_2_, *C*_i_/*C*_a_	Ratio	0.45±0.02	0.81±0.09***
∆C^18^O^16^O	‰	22.6±1.9	16.2±3.2

The plants were grown in 2% CO_2_. The measurements were made at 2% O_2_, 25 °C leaf temperature, and an irradiance of 1500 µmol quanta m^–2^ s^–1^ in a gas exchange system coupled to tuneable diode lasers that could measure either ^13^C or ^18^O discrimination. Asterisks denote statistically significant differences (*P<0.05, **P<0.01, ***P<0.001) relative to the wild type according to two-sample *t*-test. *n*=4.

### Mutation in *Sevir.5g247800,* coding for β*-*CA2, caused the *lcr* phenotype

**Fig. 6.**
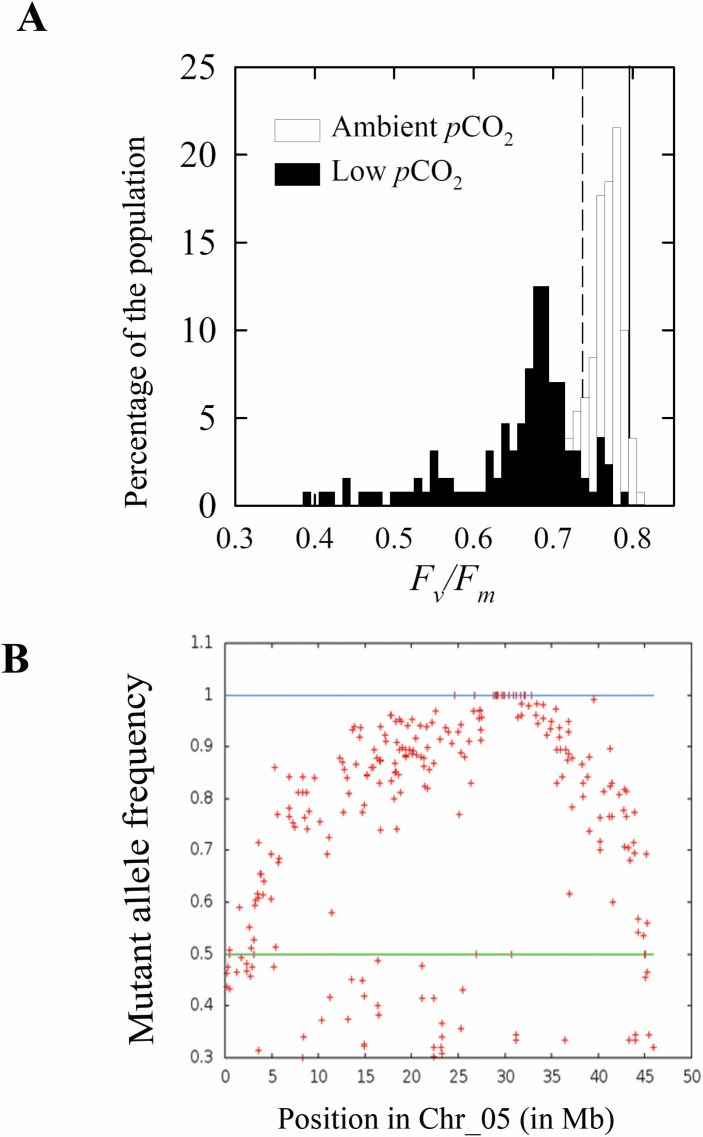
Sequencing of the BC_1_F_2_ population. (A) Histogram of *F*_v_/*F*_m_ at ambient *p*CO_2_ and after 48 h at low *p*CO_2_ in a BC_1_F_2_ population of *lcr1*. Solid and dashed vertical lines are the average *F*_v_/*F*_m_ of the wild type (WT) at ambient and low *p*CO_2_, respectively (*n*=300 for BC_1_F_2_ of *lcr1*; *n*= 50 for the WT); ~25% of the population shows mutant response. (B) Mutant allele frequencies in chromosome 5. The graph represents the plot of mutant allele frequency along the length of chromosome 5. The lines at 1 and 0.5 represent the threshold of allele frequency in the mutant and WT pool.

For identifying the causal mutation and the gene thereby affected, a BC_1_F_2_ population segregating for *F*_v_/*F*_m_ values was generated for gene mapping by sequencing (Fig. 6A). The *F*_v_/*F*_m_ values of this population ranged from 0.65 to 0.78 (median 0.78) at ambient *p*CO_2_, and from 0.39 to 0.79 (median 0.69) at low *p*CO_2_ (Fig. 6A). The percentage of BC_1_F_2_ progeny with a low *F*_v_/*F*_m_ (representing the *lcr* phenotype) was ~25% (Fig. 6A), indicative of a single recessive allele for this phenotype. Mapping-by-sequencing analysis led to a 3 Mb long region in chromosome 5, with mutant allele frequency peaking at position ~29 Mb (Fig. 6B). A total of 14 variants with alternative allele frequency of 1.0 in the mutant pool, but <0.5 in the azygous pool, were identified within this region (Supplementary Table S2). All 14 variants were single nucleotide polymorphisms (SNPs), and on functional annotation only two were found located within CDSs of genes, the rest were of low/no impact (Supplementary Table S2). Both the mutations within the gene CDSs were non-synonymous, leading to a predicted change in the encoded protein sequence. One of these mutations led to a substitution of serine to proline in a gene encoding a DNA-binding protein (*Sevir.5G251900*); as this gene is not expressed in any of the plant tissues (from public database, PhytoMine), this was not attributed as the causal mutation. The second mutation led to an amino acid change at position 156 from leucine to phenylalanine (Leu156Phe) in *Sevir.5G247800* which encodes a β-CA2 gene. Given its high expression in leaf tissues of C_4_ relatives such as maize and sorghum, it was identified as the causal gene for the *lcr* phenotype. There are two orthologues of this gene in maize (*GRMZM2G121878* and *GRMZM2G348512*), and one in sorghum (*Sobic.003G234500*). Its sorghum orthologue shows high expression in leaf tissue (Supplementary Table S3) and both the maize genes have strong differential expression between BSCs and MCs (Supplementary Fig. S5).

### Leu156Phe substitution in β*-*CA affected amino acid interactions within the protein, including new interactions with functionally important residues

The conservation profile of the amino acids in β-CA protein sequences across plant genomes showed that Leu156 is one of the 15 fully conserved amino acids (Supplementary Table S4). Eight of them were reported in the literature to play important functional roles in β-CA enzymatic activity (Kimber and Pai, 2000); Leu156, however, was not among the previously characterized residues (Supplementary Table S4). Three-dimensional structural models of WT and mutant Sevir.5G247800 (with a confidence score of 0.7) showed a characteristic dimer of β-CA, with both Leu156 and Phe156 buried inside the hydrophobic core of their respective structures, and located ~8 Å away from the catalytic site (Supplementary Fig. S6). Comparison of the CO of the mutant structure with that of the WT showed an increase in aromatic–aromatic interactions (Δ=14), but a substantial decline in aromatic–sulfur interactions (Δ= –25) (Supplementary Table S5). Leu156 interacts with some of the functionally important residues (directly or indirectly). Three such residues, present in the secondary shell of Leu156, are Gly151, Ile111, and Val170, which are involved in the active site, the catalytic cleft, and solvent access, respectively; whereas two other residues present in the tertiary shell are Cys87 and His147, both involved in ligand binding (Supplementary Table S8). The mutant protein showed new covalent interactions at primary, secondary, and tertiary interacting shells of residues to accommodate the side chain of Phe156. Some of them were unique to Phe156 interaction shells, which also included residues involved in β-CA function in plants (Supplementary Fig. S7; Supplementary Table S8). Details of the functionally important amino acids of β*-*CA are shown in Supplementary Table S9. Even the inter-monomer interactions in the homodimer were also affected in mutant dimers, with loss of seven out of 11 salt bridges (Supplementary Table S6). The possibility of a heterodimer seemed unlikely as none of the interactions of native homodimers was observed in heterodimers (Supplementary Tables S6, S7).

### Reduced abundance of CA transcript in *lcr1*

**Fig. 7.**
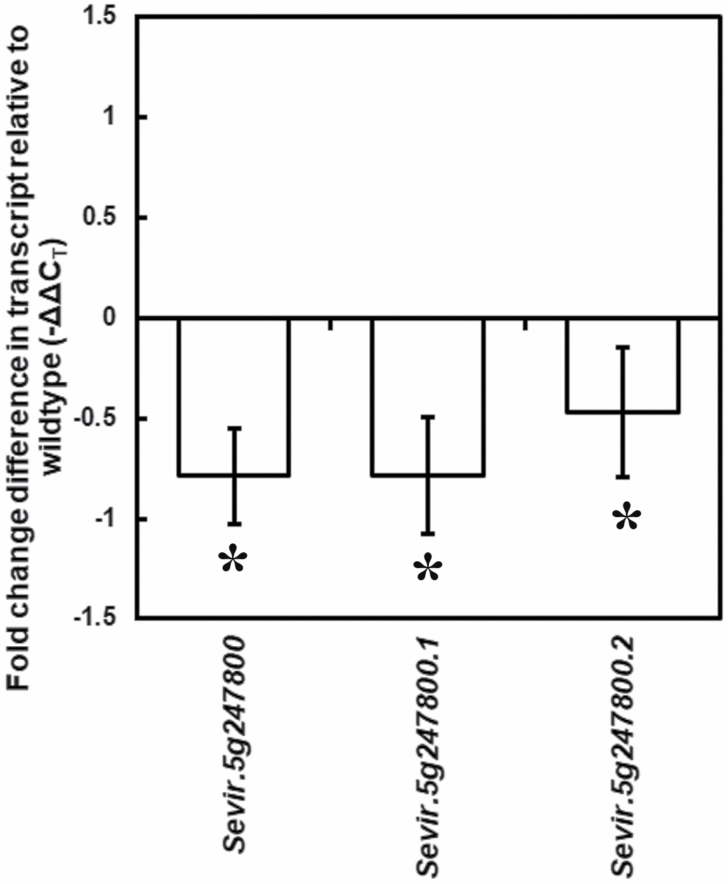
Transcript accumulation of β*-*CA spliceforms of the gene *Sevir.5g247800* as measured by quantitative real-time PCR. Values are expressed as fold changes in transcript accumulation relative to the wild type (average ±SE). Asterisks denote statistically significant differences relative to average wild type values (*P<*0.05).

To confirm that the mutation had led to suppression of β-CA transcript accumulation, quantitative real-time PCR (RT-qPCR) was performed on the mutated β-CA transcript of *lcr1.* Gene Expression Atlas data for *S. viridis* shows that Sevir.5G247800 is the most expressed β*-*CA isoform in the *S. viridis* leaf (Supplementary Fig. S8A). Expression of both the splice forms of the candidate gene (*Sevir.5g247800.1* and *Sevir.5g247800.2*) was reduced in *lcr1*, and is 0.8- and 0.5-fold lower than in WT plants (Fig. 7). Interestingly, the transcript abundance of two other tandem β-CA genes on chromosome 5 (*Sevir.5G247900* and *Sevir.5G248000*) was also reduced in *lcr1* (Supplementary Fig. S8B).

### Reduced CA activity and abundance in *lcr1*

**Fig. 8.**
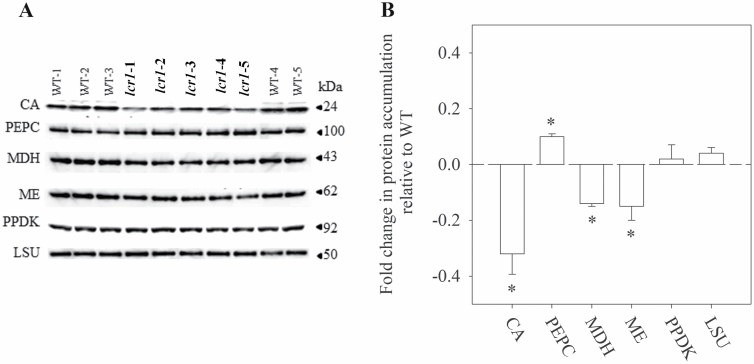
Soluble leaf proteins. (A) Western blots of wild types (WTs) and five individuals of *lcr1*. Protein was extracted from the fully expanded fourth leaf; samples were loaded on an equal leaf weight (34 µg for PEPC and LSU, 136 µg for PPDK, 340 µg for MDH, ME, and CA). (B) Integrated density values are expressed as fold changes relative to WT plants (average ±SE, *n*=5). Asterisks denote statistically significance differences relative to average WT values (*P<*0.05).

Enzyme assays revealed that the activity of CA in *lcr1* leaves was only 2% of that of the WT (Table 4). Immunoblotting with an anti-CA antibody showed an ~30% reduction in protein accumulation of CA in the mutant (Fig. 8). The abundance of primary C_4_ cycle enzymes was investigated to examine the effect of the mutation on C_4_ metabolism. The accumulation of MDH (14% lower) and ME (15% lower) was also found to be reduced in the mutant, while the accumulation of PEPC increased (10% higher) (Fig. 8). No significant differences in the accumulation of PPDK or the Rubisco LSU were observed (Fig. 8). The activities of Rubisco or PEPC (Table 4) were both significantly higher in * lcr1* (Table 4). No differences were observed in the localization of the proteins within the cellular compartments (Supplementary Figs S9–S11).

**Table 4.** Leaf biochemical properties of *lcr1*

	Units	WT	*lcr1*
Activity of C_4_ enzymes			
Carbonic anhydrase (rate constant)	mol m^–2^ s^–1^ bar^–1^	12.6±1.1	0.2±0.01***
Rubisco	µmol m^–2^ s^–1^	20.50±1.2	25.88±1.4*
PEPC	µmol m^–2^ s^–1^	166.96±7.4	221.58±10.9**

Enzyme activities were measured on 5-week-old plants grown in 2% CO_2_. The activities of carbonic anhydrase, Rubisco, and PEPC were determined as described by Osborn *et al.* (2017).

## Discussion

Carbonic anhydrase (EC 4.2.1.1) catalyses the reversible conversion of CO_2_ to HCO_3_, which is the first step of C_4_ photosynthesis (Hatch and Burnell, 1990). There are multiple families of CAs in plants, of which the β-CA isoforms are the most prevalent in higher plants (DiMario *et al.*, 2017). There are also considerable variations in CA activity between species, regardless of their photosynthetic capacities (Gillon and Yakir, 2001; Cousins *et al.*, 2008). In C_4_ species, localized within the cytosol of MCs, CA provides the substrate for PEPC (Hatch and Burnell, 1990) by accelerating the interconversion of CO_2_ to HCO_3_^–^ by up to 10^4^-fold (Badger and Price, 1994), but the importance of CA in C_4_ photosynthesis is still a matter of debate. The discrepancies among the observed phenotypes of different CA mutants have remained confusing for years. Early reports said that *in vivo* CA activity appears to be just enough to support the observed rates of photosynthesis (Jenkins *et al.*, 1989a; Hatch and Burnell; 1990), but not necessarily limiting for C_4_ photosynthesis. The growth inhibition and photosynthetic phenotype of *lcr1* is similar to that exhibited by antisense suppression of a putative cytosolic CA (CA3) in the C_4_ dicot *Flaveria bidentis*, where reduction of CA activity to 10% of that of WT plants showed marked inhibition of CO_2_ assimilation and a requirement of high CO_2_ for growth (von Caemmerer *et al.*, 2004; Cousins *et al.*, 2006). However, our results contrast with those reported for the monocot *Zea mays* (Studer *et al.*, 2014) and gene suppression work in *S. viridis* (Osborn *et al.*, 2017) in which CA activity was reduced to 50% and <13% of WT levels, respectively, but no defect in photosynthesis at ambient conditions was observed. Interestingly, in *lcr1*, mutation in a single major CA gene (*Sevir.5G247800*) was strong enough to cause a subsequent reduction in expression of the transcript of other CA isoforms too (Supplementary Fig. S8B), which possibly could have happened due to post-transcriptional silencing of a mutated copy leading to concomitant down-regulation of other homologous sequences. However, this was not observed when a major leaf CA isoform was silenced in *S. viridis* by Osborn *et al.* (2017). This could be a possible explanation for why the mutant phenotypes differed between these experiments despite the same gene being targeted.

### C_4_ phenotypic differences in *lcr1*

A reduction in CA activity would potentially limit CO_2_ hydration and the supply of bicarbonate to PEPC, leading to a distinctive photosynthetic phenotype (von Caemmerer, 2000). Consistent with this, *lcr1* exhibited severe disruptions to normal C_4_ photosynthetic function. We observed that the CO_2_ assimilation was saturated at low irradiance, and increased linearly in response to increasing intercellular *p*CO_2_. CE and quantum efficiency were significantly reduced, and the respiration rate was increased. All of these observations are indicative of a CO_2_ supply limitation, as *lcr1* recovered to some extent by application of high CO_2_. The supply of CO_2_ is definitely not hindered by the stomatal properties, as stomatal conductance (*g*_*s*_) was higher at all *p*CO_2_ in plants grown at ambient and elevated *p*CO_2_ (Supplementary Fig. S3). This increased stomatal conductance in the mutant is at odds with a typical CCM mechanism in C_4_ species which allows the leaf to function at a relatively low conductance (Jenkins *et al.*, 1989b; Brown and Byrd, 1993; He and Edwards, 1996; von Caemmerer, 2000; Kiirats *et al.*, 2002). Increased Γ is likely to be the result of a reduced CO_2_ assimilation rate relative to mitochondrial respiration as there is no increase in O_2_ sensitivity of Γ. Therefore, we concluded that the photosynthesis response in the mutant still follows a C_4_ pathway but with a much reduced assimilation rate, where the BSC permeability remains intact. The increased Δ ^13^C value in the mutants is explained rather by a change in fractionation by C_4_ fractionation factor b4, which is dependent on CO_2_ hydration (Cousins *et al.*, 2006). The Δ ^18^O decreases with reductions in CA activity (Williams *et al.*, 1996; Cousins *et al.*, 2006; Osborn *et al.*, 2017). Consistent with this, Δ ^18^O values were lower at the high *C*_i_/*C*_a_ in *lcr1* compared with the expected values in the WT (Osborn *et al.*, 2017).

### Reduction in CA affects the abundance/activity of other C_4_ enzymes in leaf

Earlier reports said that CA and PEPC may be controlled by similar regulatory mechanisms (Burnell *et al.*, 1990), and the relationship between CO_2_ hydration and PEPC carboxylation has been modelled in *Z. mays* (Farquhar, 1983). *K*_PEP_ and *K*_HCO3_ of PEPC are negatively related and linked by a conserved serine residue (DiMario and Cousins, 2019). Likewise, we have seen differences in PEPC and other C_4_ enzymes due to a change in behaviour of the *lcr1* gene. There was an apparent increase in PEPC protein abundance (Fig. 8), possibly due to a feedback response with a significant change in PEPC activity (Table 4). Interestingly, the Rubisco activity is also significantly increased in the mutant. Generally, in C_4_ plants, changes in CE are considered to be mostly related to PEPC activity assuming an excess of CA and saturated rates of Rubisco activity or rates of ribulose bisphosphate regeneration (Berry and Farquhar, 1978; Peisker, 1979; Collatz *et al.*, 1992; von Caemmerer and Furbank, 1999). However, our result suggests that the reduction in CA activity in *lcr1* is strong enough to alter the normal photosynthetic machinery in *lcr1*.

### A single amino acid change (Leu156Phe) in Sevir.5G247800 creates structural and functional alterations in the protein

In *S. viridis* there are four genes encoding β*-*CA genes, CA5 (*Sevir.2G245200*) located on chromosome 2, two CA2 (*Sevir.5G247800* and *Sevir.5G247900*) and CA4 (*Sevir.5G248000*) located side by side on chromosome 5. There are two isoforms of the mutated gene (*Sevir.5g24780*; CA2) (Christin and Osborne, 2013; John *et al.*, 2014) as also confirmed from our transcript analysis (Supplementary Fig. S8). All the genes are expressed in the cytosol of the M cells. Sequencing of *lcr1* led to non-synonymous substitution from a leucine residue (L156) to a phenylalanine residue in the major β*-*CA gene *Sevir.5g248000*. Although the functional role of Leu156 was not reported by Kimber and Pai (2000), its complete conservation in the angiosperm β-CA family (Supplementary Table S4) indicates a plant-specific role. Due to the proximity of Leu156 to the active site (~8 Å), and even the (hydrophobic) residues lining the narrow channel through which solvent reaches the active site, it interacts with functionally important residues in the protein. Its substitution by phenylalanine altered the existing interactions (Supplementary Fig. S7; Supplementary Table S8), resulting in possible functional perturbation. Additionally, the positional and/or physicochemical nature of Leu156 indirectly played an important role in maintenance of aromatic–aromatic interactions within the β*-*CA protein structure. The substitution by phenylalanine, an aromatic amino acid, substantially altered the existing aromatic–aromatic interactions, such that there was a large reduction in CO within the mutant protein with respect to the native protein (Supplementary Table S5), which seemed to negatively impact the structural stability of the protein, and eventually the normal functioning of the β*-*CA enzyme. The mutation even alters transcript accumulation for all CA genes located on chromosome 5 and a significant reduction in total leaf CA protein abundance and activity (Supplementary Fig. S8; Fig. 8; Table 4).

### *Sevir.5g247800* as a target to achieve CA response in C_3_ plans

CA in C_3_ plants has similar kinetic properties to those of C_4_ plants (Hatch and Burnell, 1990), but it is localized in a different subcellular compartment (Tanz *et al.*, 2009). Evolutionary studies predicted that the C_4_ CA has evolved from C_3_ CA by loss of transit peptide and relocation of the enzyme in the cytosol; eventually, it was recruited in the C_4_-specific carboxylation function (Ludwig, 2012). It is predicted that the most abundant CA in the C_3_ chloroplast stroma was actually modified in this way and present in multiple copies in C_4_ species. Evidence from the phylogeny study shows that rice homologues *LOC_Os01g45274* and *LOC_Os09g28910* are the closest homologues of *Sevir.5G247800*, with 83.3.% and 48.0% protein similarity, respectively. Both of these rice genes code for chloroplastic precursors of the β-CA gene in rice and might be potential targets to create a CA response in rice. On this note, the transgenic approach is adopted by the C_4_ consortium to overexpress the C_4_ CA from *Z. mays* in the MCs of rice using a cell-specific promoter (Kajala *et al.*, 2011). However, it still remains unclear if the multiple roles for CA in C_3_ plants are an important consideration for manipulating the accumulation and activity of these proteins in C_3_ plants. Therefore, given the involvement of a single CA isoform in the C_4_ pathway in *S. viridis*, it may be a better choice than the CA of *Z.mays*, and can be incorporated in a C_3_ species along with other C_4_ enzymes to create a C_4_ environment. Even the *cis-*element of *Sevir.5G247800* could be tested for C_4_-specific gene expression in the mesophyll cytosol.

Overall, our results demonstrate unequivocally that CA is an absolute requirement for C_4_ photosynthesis, and that uncatalysed rates of CO_2_ to HCO_3_^–^ appear to be insufficient to support C_4_ photosynthetic flux (Hatch and Burnell, 1990; Badger and Price, 1994). Our results highlight the potential importance of expressing at least some CA in the mesophyll cytosol of rice MCs in order to generate a functional C_4_ biochemical pathway capable of high rates of photosynthesis.

### Conclusion

Our results highlight the potential utility of chemical mutant screens to identify key genes involved in C_4_ photosynthesis. The mutant shows a large reduction in carbon assimilation, a higher CO_2_ compensation point, and higher carbon isotope discrimination. Analysis reveals mutation in the β*-*CA gene in the mutant, which not only causes a large reduction in CA activity in the, but also changes C_4_ enzyme abundance. This study concludes that despite conflicting evidence, CA is a crucial first step in the C_4_ pathway, at least in *S. viridis*, and potentially in all C_4_ plants. In other species, different CA isoforms may take on this role and be responsible for a similar phenotype.

## Supplementary data

The following supplementary data are available at *JXB* online.

Table S1. Stomatal properties of *lcr1* at elevated CO_2_ level 2.

Table S2. SNPs associated with the *lcr* phenotype.

Table S3. Sorghum β-CA gene *Sobic.003G234500* expression in leaf, extracted from Phytomine (https://phytozome.jgi.doe.gov/phytomine/report.do?id=358824185#expression).

Table S4. List of amino acid residues found to be fully conserved in β-CA homologues across entire plant genomes.

Table S5. Contact order values for each interaction type in native and mutant structures.

Table S6. Change in inter-monomer interactions between native and mutant structures of β-CA.

Table S7. Change in inter-monomer interactions between native (homodimer) and isoform heterodimer structures of β-CA.

Table S8. List of amino acid residues found in primary, secondary, and tertiary shells of Leu156 and Phe156.

Table S9. List of functionally important amino acid residues identified in *Pisum sativum* β-CA by Kimber and Pai (2000) and Rowlett (2010).

Fig. S1. Recovery of leaf anatomical structure in elevated CO_2_.

Fig. S2. Rapid chlorophyll fluorescence kinetics.

Fig. S3. Stomatal conductance (*g*_s_) of *lcr1* and the WT in ambient and elevated CO_2_ conditions.

Fig. S4. Net CO_2_ assimilation rate (*A*) at different oxygen levels.

Fig. S5. Expression pattern in leaf developmental gradient of maize β-CA2 (*Sevir.5G247800*) orthologues GRMZM2G121878 and GRMZM2G348512.

Fig. S6. Homology models of native and mutant Sevir.5G247800 protein structures.

Fig. S7. Venn diagram comparing combined interaction shells (primary, secondary, and tertiary) of Leu156 and Phe156, and further with the functionally important residues of β-CA (as per Kimber and Pai, 2000).

Fig. S8. Expression pattern of β-CA isoforms in the Gene Expression Atlas of *S. viridis* (A) and on quantification by RT-qPCR in *lcr1* (B).

Fig. S9. Representative images of immunolocalization of carbonic anhydrase protein in wild-type and M_6_ generation *lcr1* plants.

Fig. S10. Representative images of immunolocalization of Rubisco protein in wild-type and M_5_ generation *lcr1* plants.

Fig. S11. Representative images of immunolocalization of PEPC protein in wild-type and M_6_ generation *lcr1* plants.

erab039_suppl_Supplementary_Tables_S1-S9_and_Figures_S1-S11

## Data Availability

The raw reads from whole genome sequencing of pooled samples used in this work is available at NCBI’s Short Read Archive (SRA) under Bioproject ID PRJNA692561 (experiment: SRX10094751).

## Abbreviations

A: net rate of CO_2_ assimilation
CA: carbonic anhydrase
CCM: carbon-concentrating mechanism
CE: maximum carboxylation efficiency
*C*_i_: intercellular CO_2_ concentration
*F*_v_/*F*_m_: maximum quantum efficiency of PSII photochemistry
*F*_v_′/*F*_m_′: maximum efficiency of PSII in the light
lcr: *low CO*_*2*_ *-responsive mutant*
LSU: large subunit of Rubisco
MDH: malate dehydrogenase
ME: malic enzyme
NMU: *N*-nitroso-*N*-methylurea
NPQ: non-photochemical quenching
PEPC: phospho*enol*pyruvate carboxylase
PPDK: pyruvate phosphate dikinase
qP: fraction of the maximum PSII efficiency that is realized in the light
PPFD: photosynthetic photon flux density
*R*_d_: dark respiration rates
qP: photochemical quenching
QY: quantum yield
Γ: CO_2_ compensation point
Φ: quantum yield for CO_2_ assimilation
ΦPSII: PSII operating efficiency
