# Supplementary material for: A low CO2-responsive mutant of Setaria viridis reveals that reduced carbonic anhydrase limits C4 photosynthesis
Source: J Exp Bot. 2021 Feb 2;72(8):3122–36. doi: 10.1093/jxb/erab039 (PMC8023212; doi:10.1093/jxb/erab039)
Supplement: erab039_suppl_Supplementary_Tables_S1-S9_and_Figures_S1-S11 [file erab039_suppl_supplementary_tables_s1-s9_and_figures_s1-s11.pdf]

Table S1: Stomatal properties of *lcr1* at elevated CO_2 level 2.

**Stomatal properties**	**Unit**	**WT**	***lcr1***
Stomatal density, n=20	no. mm^{-2}	122.5 ± 2.91	124.0 ± 2.28
Stomatal height, n=164	µm	27.3 ± 0.23	27.9 ± 0.20
Stomatal width, n=164	µm	22.2 ± 0.26	21.3 ± 0.19**
Stomatal area, n=164	$µm^2$	453.3 ± 7.29	453.1 ± 5.04

Stomatal measurements were performed on the abaxial side of the leaf of 5-week old plants grown in 2% CO_2. Stomatal number was counted from 5 images taken from each plant, and the stomatal height, width and area were measured from 164 random stomata taken from these 5 images.

Table S2. SNPs associated with the *lcr* phenotype. These SNPs had alternate allele frequency of 1 in mutant pool, and were located within Chromosome 5:28,736,238 - 31,681,508. Annotation of the SNPs examined impact of the mutation on gene function.

#	Position (Mb)	SNP	Mutant allele frequency	Azygous allele frequency	Gene/location in genome	Impact
1	28,736,238	C/T	1	0.20	Intergenic	Non significant
2	28,794,999	C/T	1	0.28	Intron of Sevir.5G239700	Non significant
3	28,991,394	C/T	1	0.20	Intergenic	Non significant
4	29,104,173	A/G	1	0.28	Intergenic	Non significant
5	29,166,852	C/T	1	0.26	No gene there but it is homologous to rice transposon protein	Non significant
6	29,237,092	C/T	1	0.259	Intergenic	Non significant
7	29,287,560	T/C	1	0.20	Intergenic	Non significant
8	29,681,012	C/T	1	0.27	Sevir.5G247800 (Carbonic anhydrase)	Amino acid change (Leu → Phe)
9	29,814,037	C/T	1	0.145	Intergenic	Non significant
10	29,979,934	C/T	1	0.20	Intron of Sevir.5G251800	Non significant
11	29,985,238	A/G	1	0.20	Coding region of Sevir.5G251900 (DNA binding protein)	Amino acid change (Ser → Pro)
12	30,419,734	C/T	1	0.25	Intergenic	Non significant
13	30,939,701	C/T	1	0.24	Intergenic	Non significant
14	31,681,508	A/G	1	0.50	No gene there but it is homologous to an EST	Non significant

Table S3: Sorghum *β*-CA gene *Sobic.003G234500* expression in leaf , extracted from Phytomine (https://phytozome.jgi.doe.gov/phytomine/report.do?id=358824185#expression)

**Part of the plant.Deveopmental stage**	**Experimental group**	**Transcript abundance (FPKM)**
Leaf lower whorl.Vegetative	GeneAtlas Tissue Sample	0.6
Leaf sheath growing.Floral initiation	GeneAtlas Tissue Sample	0.9
leaf flag 1 internode.Anthesis	GeneAtlas Tissue Sample	1.7
leaf flag 1 internode.Grain maturity	GeneAtlas Tissue Sample	2.3
Leaf lower.Juvenile	GeneAtlas Tissue Sample	2.3
Leaf sheath growing.Grain maturity	GeneAtlas Tissue Sample	3.3
Leaf middle whorl.Vegetative	GeneAtlas Tissue Sample	3.5
Leaf upper.Juvenile	GeneAtlas Tissue Sample	4.2
Leaf sheath growing.Anthesis	GeneAtlas Tissue Sample	5.5
Leaf blade.Juvenile	GeneAtlas Tissue Sample	7.2
Leaf upper whorl.Vegetative	GeneAtlas Tissue Sample	7.4
Leaf upper growing.Floral initiation	GeneAtlas Tissue Sample	8.1
Leaf lower growing.Grain maturity	GeneAtlas Tissue Sample	8.9
Leaf lower growing.Floral initiation	GeneAtlas Tissue Sample	9.0
Leaf lower growing.Anthesis	GeneAtlas Tissue Sample	12.7
Leaf upper growing.Anthesis	GeneAtlas Tissue Sample	14.4

Table S4. List of amino acid residues found fully-conserved in beta-CA homologs across entire plant genomes. Those reported in literature for functional role have also been mentioned. The positions are with respect to Sevir.5G247800 peptide sequence. CA2' gene family from Angiosperms were obtained from Phytozome database (https://phytozome.jgi.doe.gov)

**S. No.**	**Residue with position**	**Functional role (as in Kimber & Pai, 2000)**
1	Cys87	Zn ligand binding
2	Asp89	Zn ligand binding
3	Ser90	Active site
4	Arg91	Active site
5	Gly103	Not reported
6	Phe106	Active site cleft
7	Arg109	Non-catalytic bicarbonate binding pocket
8	Ala112	-Not reported
9	Gly146	-Not reported
10	His147	Zn ligand binding
11	Cys150	Zn ligand binding
12	Gly152	Not reported
13	Ile153	Not reported
14	Leu156	Not reported
15	Trp173	Not reported

Table S5. Contact order values for each interaction type in native and mutant structures.

Interactions	Native	Mutant	Change
Hydrophobic	29.259	28.758	-0.5
Main chain- Main chain hydrogen bonds	7.043	6.913	-0.13
Main chain - Side chain hydrogen bonds	22.992	23.043	0.05
Side chain - Side chain hydrogen bonds	21.115	26.319	5.2
Ionic bonds	18.156	19.853	1.7
Aromatic -Aromatic interactions	28.333	42.429	14
Aromatic - Sulfur interactions	55.500	30.222	-25.3
Cation - Pi interactions	38.000	42.857	4.86

Table S6. Change in inter-monomer interactions between native and mutant structures of beta-CA.

Interaction type	Common (to both native and mutant)	Native-specific	Mutant-specific	Total
Hydrogen bonds	16	15	24	55
Non-bonded contacts	115	89	78	300
Salt bridges	2	7	2	11

Table S7. Change in inter-monomer interactions between native (homo-dimer) and isoform hetero-dimer structures of beta-CA.

Interaction type	Common (to both)	Native homo-dimer specific	Isoform hetero-dimer specific	Total
Hydrogen bonds	0	31	24	56
Non-bonded contacts	0	204	171	375
Salt bridges	0	9	6	15

Table S8. List of amino acid residues found in primary, secondary and tertiary shells of Leu156 and Phe156. The residue numbers in yellow, green and blue colors are those functionally important residues form Kimber and Pai (2000), which are specific to Phe156's shell, having shifted to inner shells of Phe156 w.r.t. Leu156, and common to both Leu156 & Phe156 shells, respectively.

**Leu156**			**Phe156**		
**primary**	**secondary**	**tertiary**	**primary**	**secondary**	**tertiary**
114, 152, 153, 154, 158, 174, 180, 181, 200	**111**, 112, 150, **151**, 155, 156, 157, 159, 160, **170**, 171, 173, 176, 177, 178, 179, 183, 184, 185, 196, 197, 198, 202, 203, 204, 207	**87**, 110, 113, 116, 117, 123, 126, **147**, 148, 149, 162, 164, 166, 167, 169, 172, 175, 180, 182, 186, 187, 188, 189, 191, 192, 193, 194, 195, 199, 201, 205, 206, 208, 209, 210, 211, 293	**111**, 112, 114, 152, 153, 154, 157, 173, 174, 181, 200, 204	**87**, 110, 113, 116, 123, 150, **151**, 155, 158, 169, **170**, 172, 175, 176, 177, 178, 179, 180, 183, 184, 185, 193, 194, 196, 197, 198, 201, 202, 203, 207, 208	85, 86, 88, 89, 109, 115, 117, 119, 122, 125, 126, 127, 144, **147**, 148, 149, 159, 160, 167, 169, 171, 182, 186, 187, 188, 189, 191, 192, 194, 195, 199, 201, 205, 206, 209, 210, 211, 212, 215, 233

Table S9. List of functionally important amino acid residues identified in *Pisum sativum* beta-CA by Kimber and Pai (2000), the same also listed in Rowett (2010). The positions mapped in Sevir.5G247800 protein are also listed. The prime symbol (’) indicates residues of another monomer of the dimer.

**Active site**		**Active site cleft**		**Ligand binding**		**Solvent access**		**Non-catalytic HCO3-- binding pocket**	
P sativum	***S. viridis***	***P sativum***	***S. viridis***	***P sativum***	***S. viridis***	***P sativum***	***S. viridis***	***P sativum***	***S. viridis***
Q151’	Q78’	F179’	F106’	C160	C87	Tyr205’	Tyr132’	V157	V84
D162	D89	V184	I111	D162	D89	Gln151’	Q78’	A159	A86
S163	S90	Y205’	Y132’	H220	H147	Gly224	Gly151	V165	C92
R164	R91	H209’	A136’	C150	C223	Asp162	Asp89	R182	R109
G224	G151					Ile243	Val170	Y307	Y234

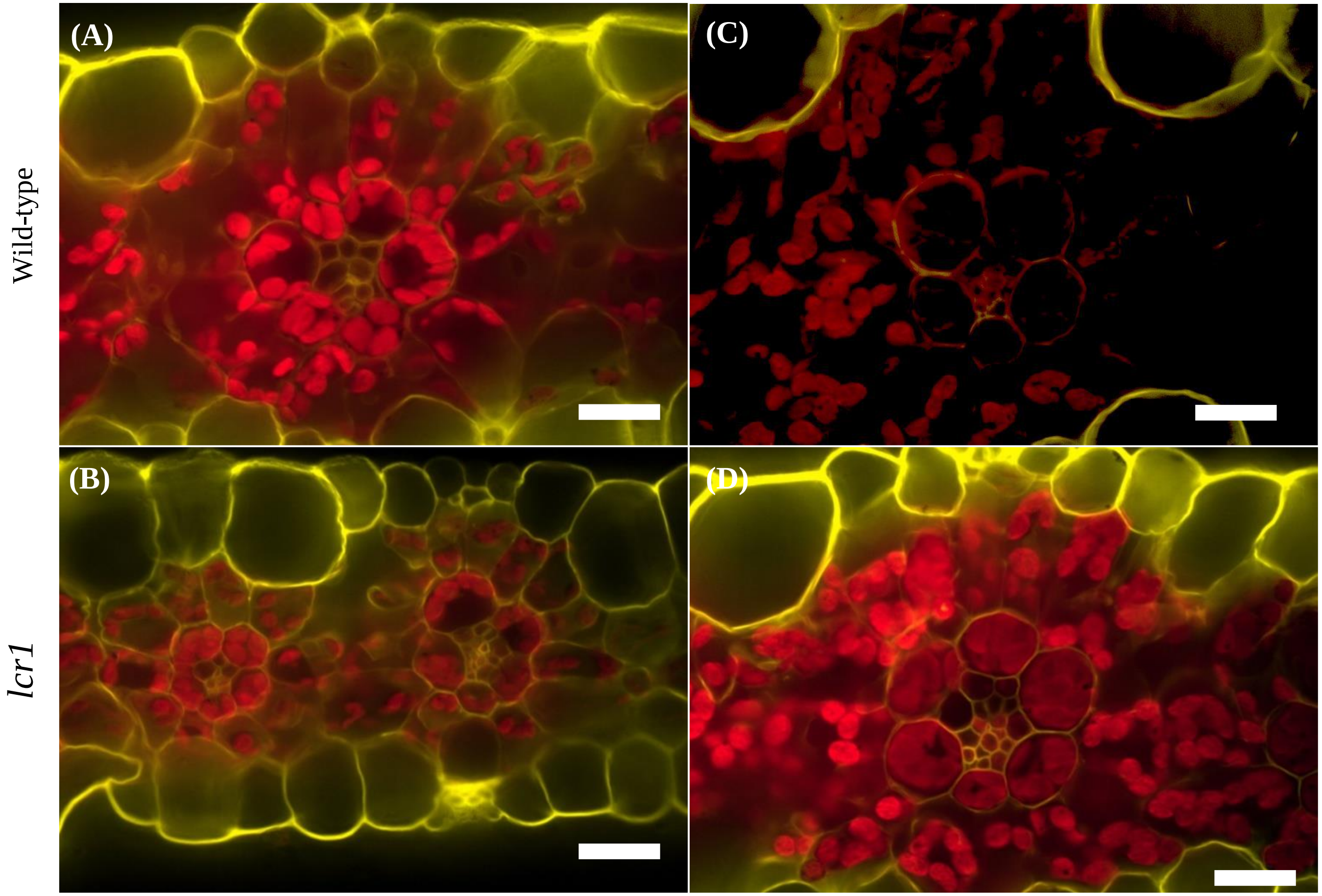

Fig. S1. Recovery of leaf anatomical structure in elevated CO_2. Representative Fluorescent images of transverse leaf sections of wild-type (**A**,C) and M_6 generation *lcr1* (B,D) leaves collected in the afternoon from plants grown at (**A**,B) ambient pCO_2 and (**C, D**) elevated pCO_2 (10,000 µbar). Images acquired at the middle of the leaves. Scale bar 20 µm. Yellow shows the cell wall , red shows the auto-fluorescence of chloroplast in mesophyll and bundle sheath cells.

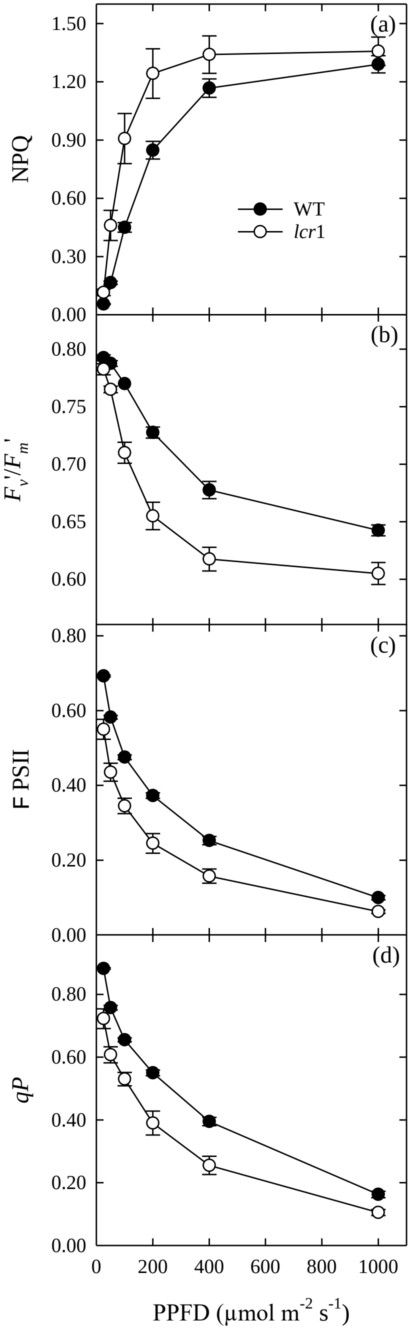

Fig. S2. Rapid chlorophyll fluorescence kinetics. Response of (a) non-photochemical quenching (NPQ), (b) maximum quantum efficiency of PSII (F_v'/F_m'), (c) PSII operating efficiency (φPSII), (d) the fraction of the maximum PSII efficiency that is realized in the light (*qP*) in wild-type (WT) and M_5 generation *lcr1* plants to increasing photosynthetically active photon flux density (PPFD). Values are the average ± SE. of 9 individual plants per line grown at ambient pCO_2. **This figure is redrawn from Coe et al., 2018.**

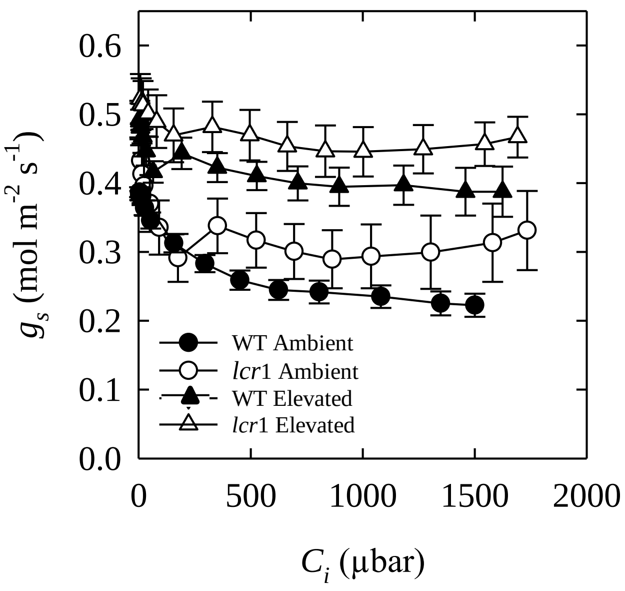

Fig. S3 . Stomatal conductance (g_s) of *lcr1* and WT in ambient and elevated CO_2 condition. Measurements were taken at constant 1800 PPFD at different CO_2 levels using infrared gas analyzer at leaf temperature of 30ºC. Error bars are ± SE of three leaves from three plants.

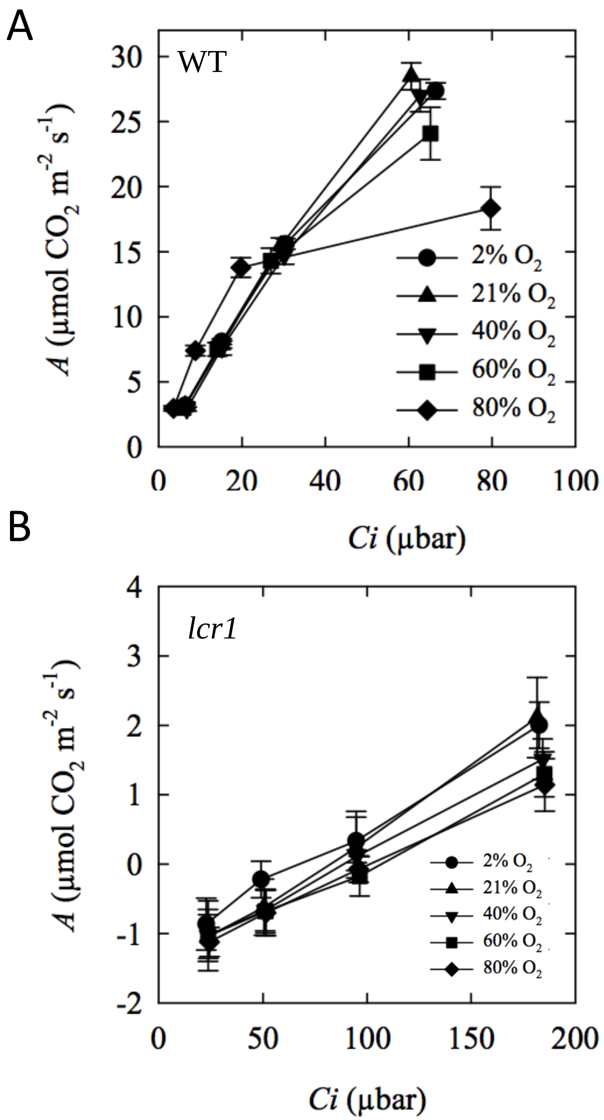

Fig. S4. Net CO_2 assimilation rate (*A*) in response to changes in intercellular pCO_2 (*Ci*) at 2-80% O_2 in (A) wild-type and (B) M_5 generation *lcr1* plants. Measurements were made at 1800 µmol photons m^{-2} s^{-1} and a leaf temperature of 30 °C. Values represent the mean ± SE of 1 leaf from 3 plants. **O_2 concentration in the IRGA was maintained by mixing O_2 and N_2 gas from a cylinder and supplied via an overflow tube to the air inlet of the Li-Cor and blended with CO_2.**

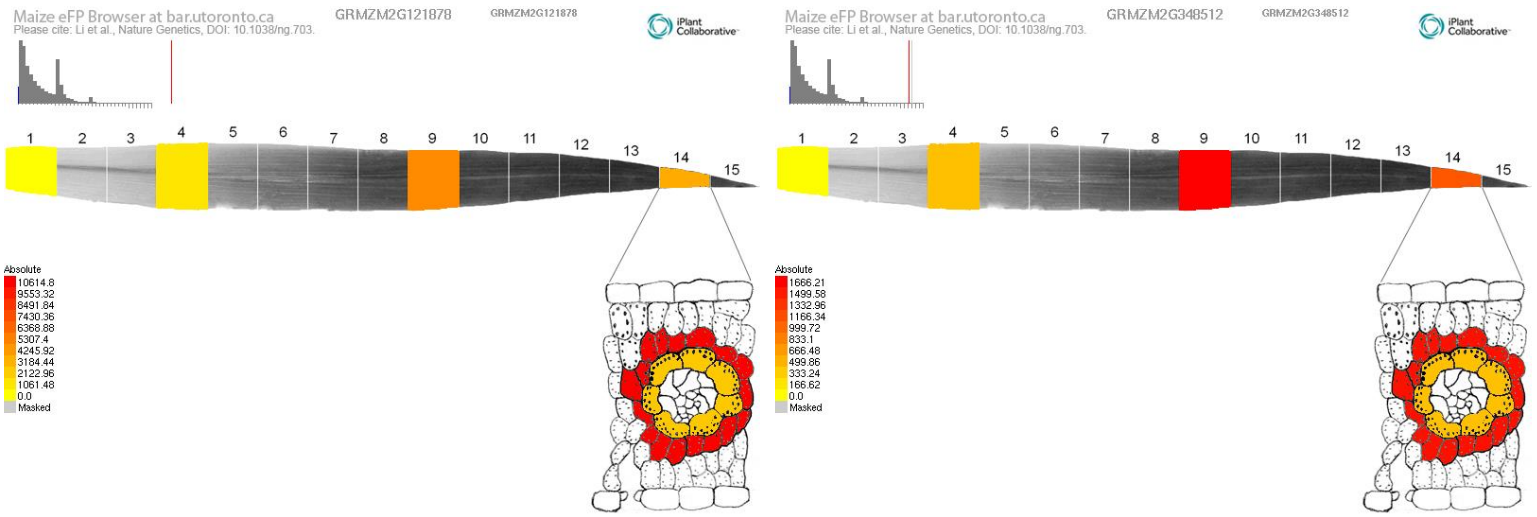

Fig. S5. Expression pattern in leaf developmental gradient of maize β-CA2 orthologs GRMZM2G121878 and GRMZM2G348512. The expression data was obtained from Maize eFP browser (www.bar.utoronto.ca).

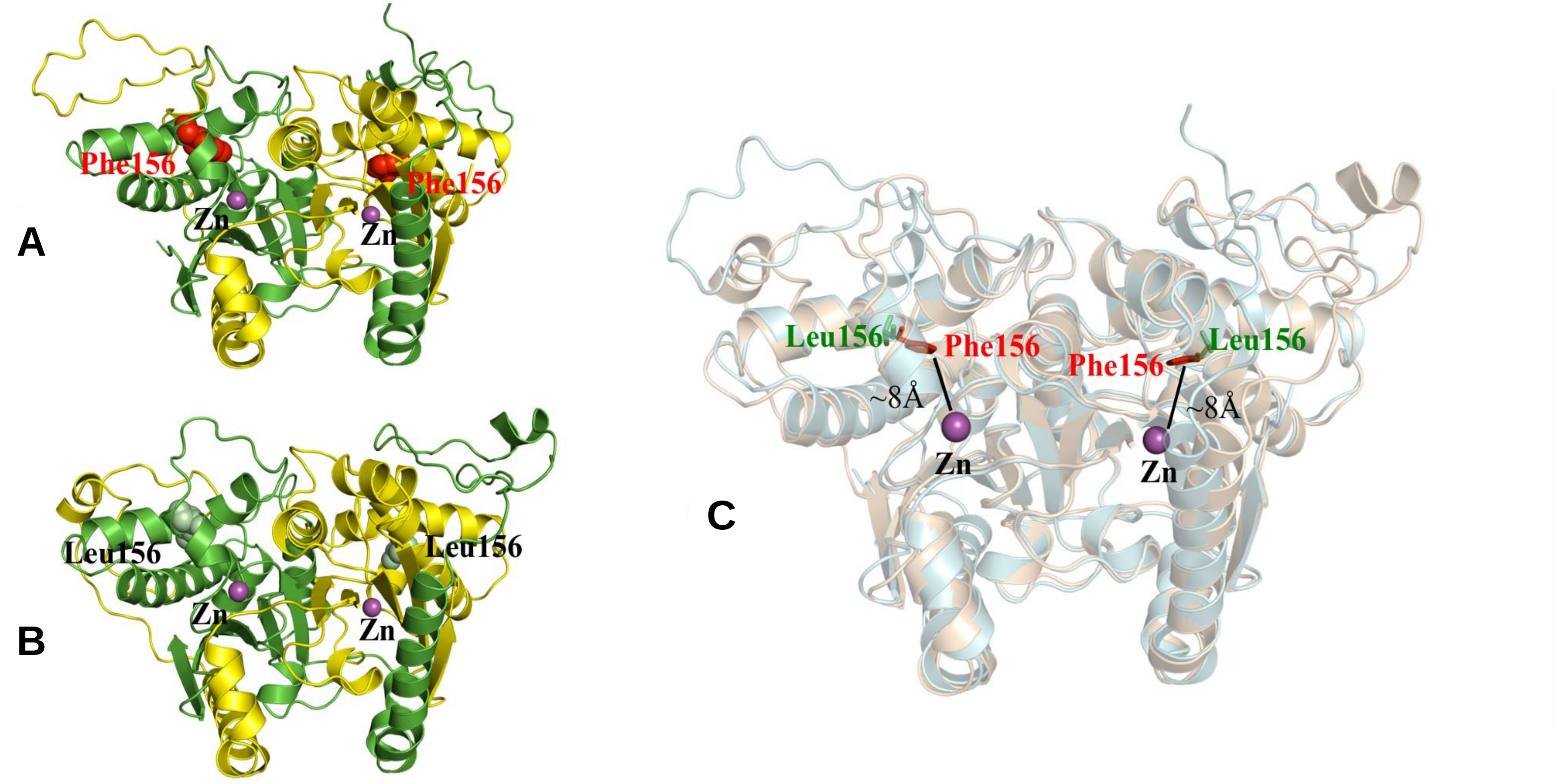

Fig. S6. **Homology models of native and mutant Sevir.5G247800 protein structures**. The cartoon representation of the mutant (a), native (b), and superimposed native and mutant structures (c) are shown. Since the *β*-CA functions in dimer or in other higher oligomeric forms, so the dimeric form model of Sevir.5G247800 was considered; the monomers are indicated in different colors (green and yellow) in the subfigures (A) and (B). The amino acid residue of interest (Leu156 or Phe156) are shown as red or grey spheres, respectively. The location of Zn ion is also highlighted in all subfigures, and the distance between the ion and the substituted residue is also shown in (C).

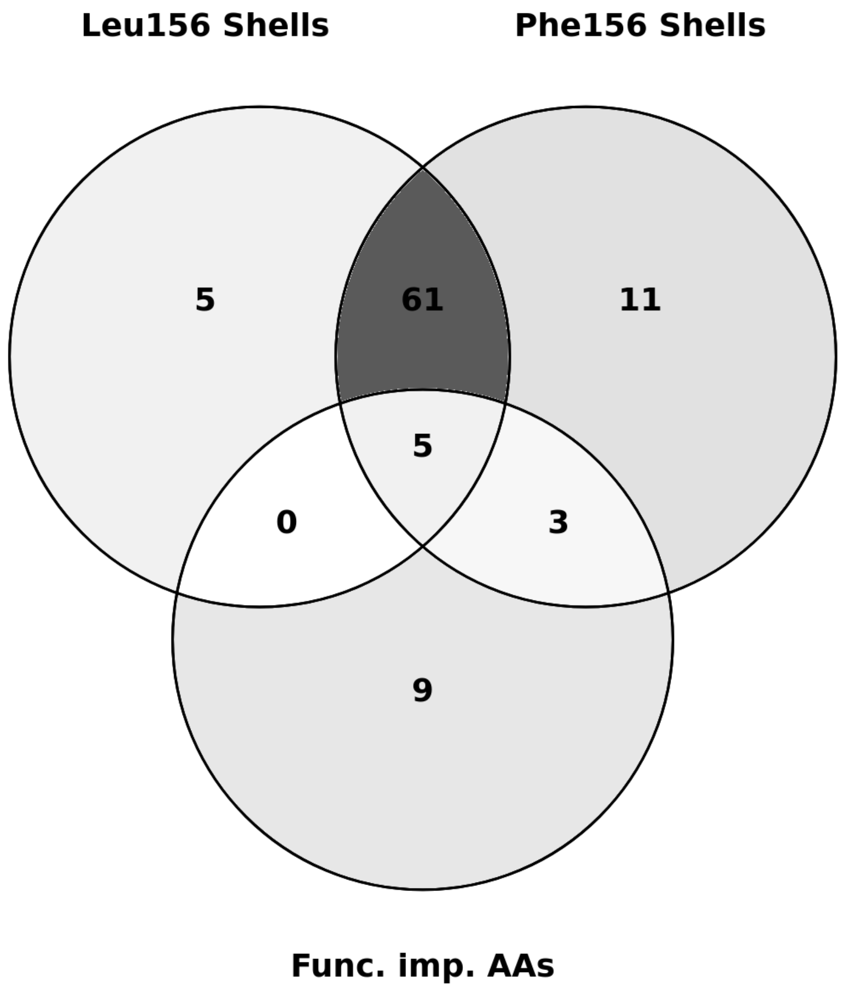

Fig. S7. Venn diagram comparing combined interaction of primary, secondary and tertiary shells of Leu156 and Phe156, and further with the functionally important residues of beta-CA (as per Kimber and Pai, 2000). Three functionally important residues, which were also specific to Phe156 shells, were: Ala86, Asp89 and Arg109. While Asp89 was involved in Zn ligand binding, the other two are involved in forming non-catalytic bicarbonate binding pocket. Among five functionally important residues, which were common to both shells, two of them shifted to inner interaction shell of Phe156 (Cys87 and Ile111), and are involved in Zn ligand binding and part of active site cleft, respectively. The intensity of the colors represents the relative numbers.

(A)

(B)

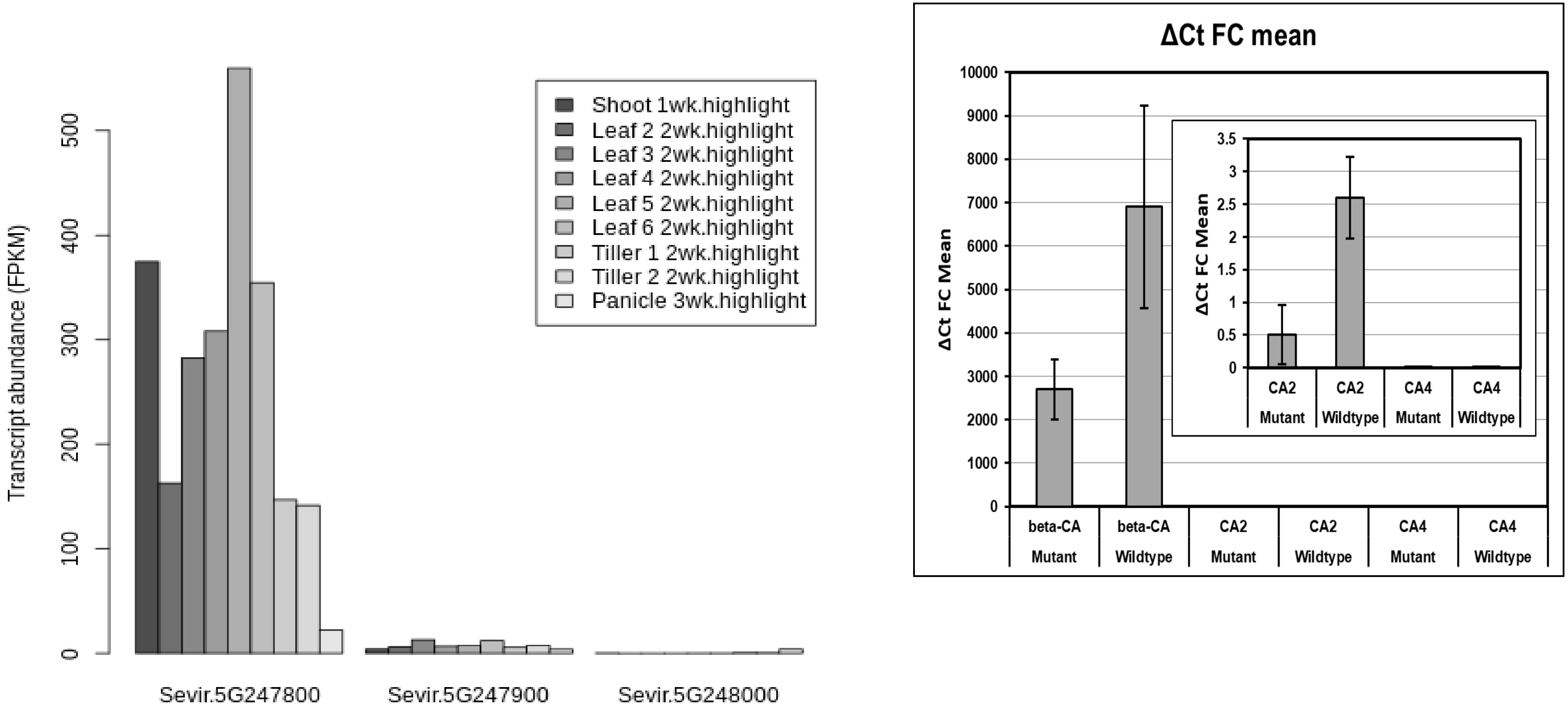

Fig. S8. Expression pattern of βCA isoforms in *S. viridis* gene expression atlas (A) by RT-qPCR in *lcr1* (B). The gene expression data shown in (A) was obtained from 'Expression data sources' of Phytomine/Phytozome database. In (B), expression of both βCA2 (*Sevir. 5g247800* and *Sevir. 5g247900*) and CA4 (*Sevir.5g248000*) was reduced compared to WT Setaria in RT-qPCR based expression analysis.

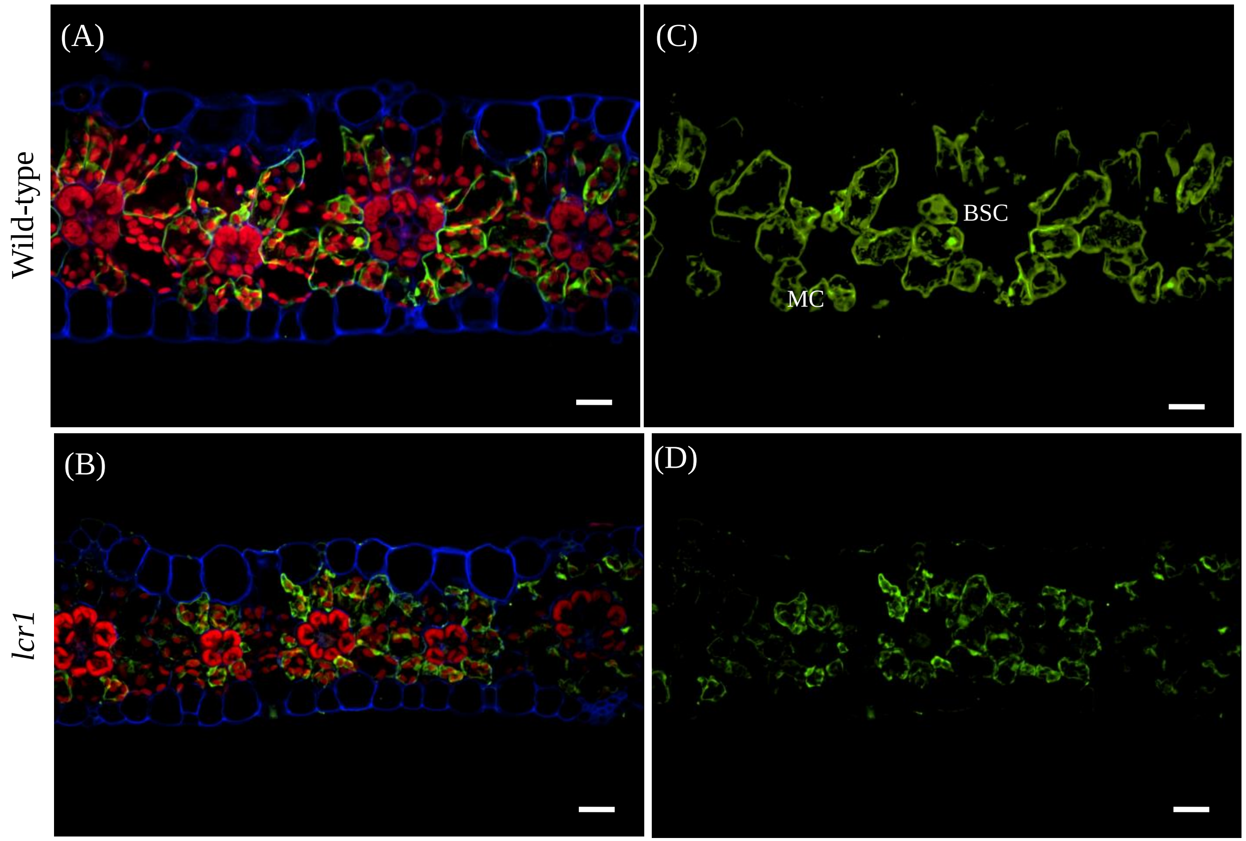

Fig. S9. Representative images of immunolocalization of carbonic anhydrase protein in wild-type (**A**,**C**) and M_6 generation *lcr1* (B,D) plants. Anti-CA rabbit polyclonal primary antibody (1:50) plus Alexa Fluor 488 goat anti-rabbit IgG as secondary antibody (1:200; shown in green color). Red shows autofluorescence of chlorophyll in chloroplast. Co-staining with calcofluor white visualized cell wall (shown in blue). Green shows the presence of CA in leaf. Magnification: 200x. Scale bar: 20 µm. Images are of the middle portion the fifth fully expanded fifth leaf. MC = mesophyll cells, BSC = Bundle sheath cells.

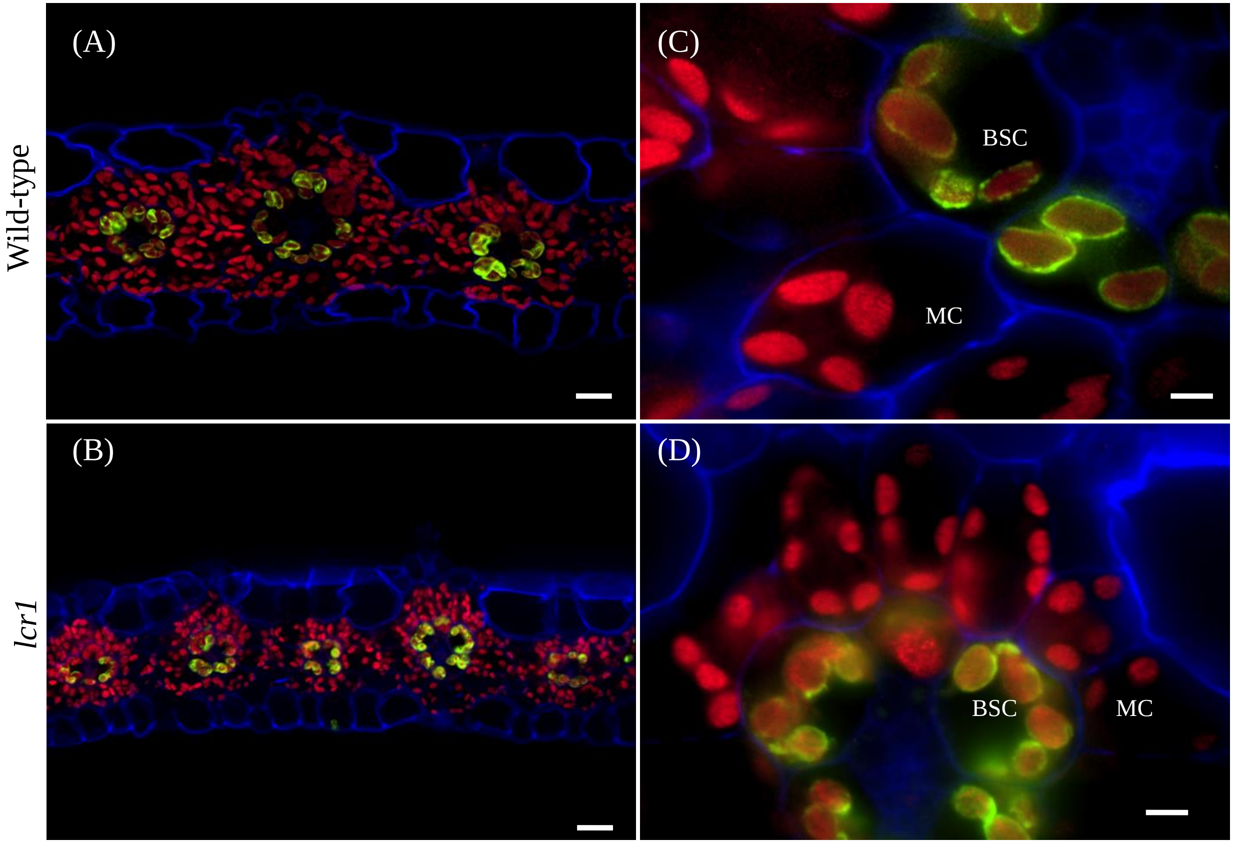

Fig. S10. Representative images of immunolocalization of Rubisco protein in wild-type (**A**,C) and M_5 generation *lcr1* (**B**,D) plants. Anti-Rubisco rabbit polyclonal primary antibody (1:100) plus Alexa Fluor 488 goat anti-rabbit IgG as secondary antibody (1:200; shown in green color). Red shows autofluorescence of chlorophyll in chloroplast. Co-staining with calcofluor white visualized cell wall (shown in blue). Green shows the presence of Rubisco in leaf. Magnification: 200x (**A**,B) and 100x (**C**,D). Scale bar: 20 (**A**,B) or 5 (**C**,D) µm. Images are of the middle portion the fifth fully expanded fifth leaf. MC = mesophyll cells, BSC = bundle sheath cells.

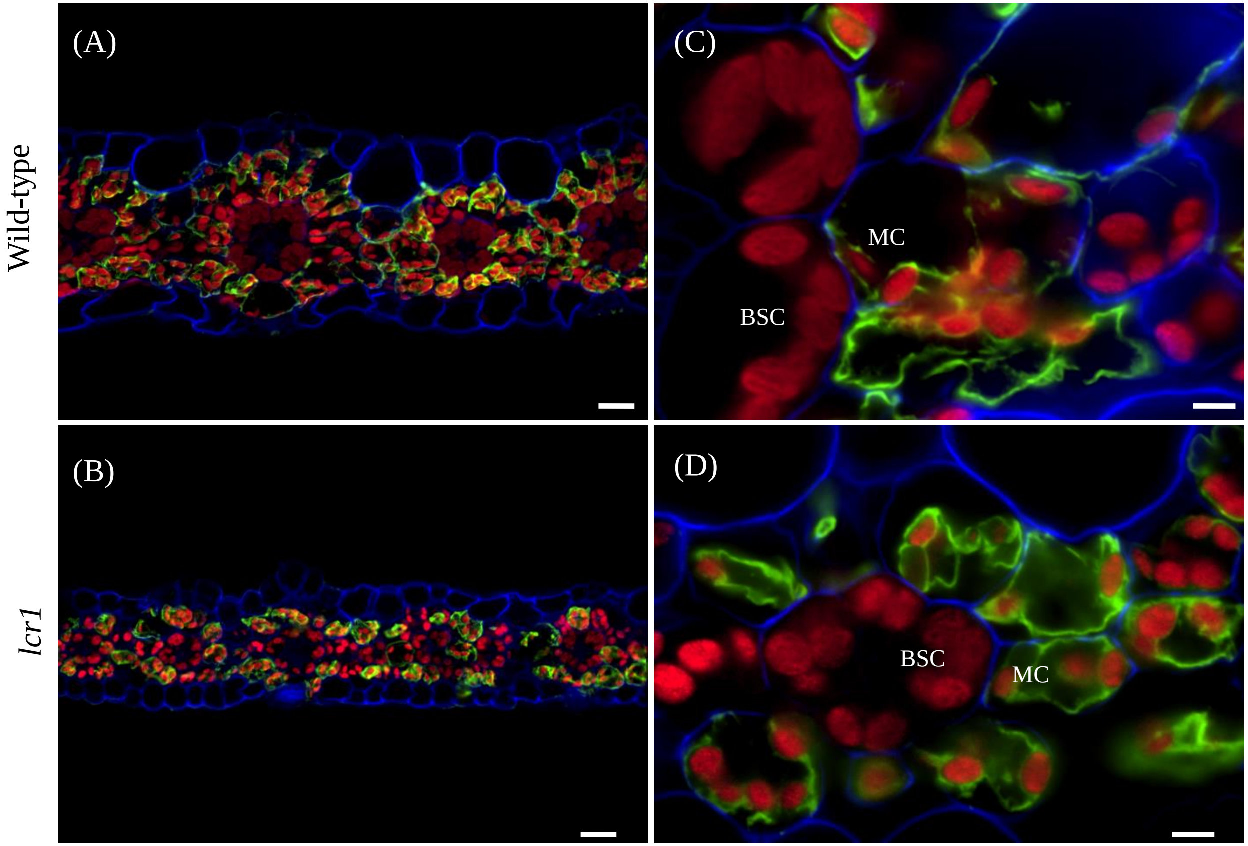

Fig. S11. Representative images of immunolocalization of PEPC protein in wild-type (A,C) and M_6 generation *lcr1* (B,D) plants. Anti-PEPC rabbit polyclonal primary antibody (1:100) plus Alexa Fluor 488 goat anti-rabbit IgG as secondary antibody (1:200; shown in green colour). Red shows autofluorescence of chlorophyll in chloroplast. Co-staining with calcofluor white visualized cell wall (shown in blue). Green shows the presence of PEPC in leaf. Magnification: 200x (A,B) and 1000x (C.D). Scale bar: 20 (A,B) or 5 (C,D) µm. Images are of the middle portion of the fifth fully expanded leaf. MC = mesophyll cells, BSC = Bundle sheath cells.
